# Glasdegib Dimaleate: Synthesis, Characterization and Comparison of Its Properties with Monomaleate Analogue

**DOI:** 10.3390/pharmaceutics14081641

**Published:** 2022-08-06

**Authors:** Boris Peklar, Franc Perdih, Damjan Makuc, Janez Plavec, Jérôme Cluzeau, Zoran Kitanovski, Zdenko Časar

**Affiliations:** 1 Lek Pharmaceuticals d.d., Sandoz Development Center Slovenia, Kolodvorska 27, 1234 Mengeš, Slovenia; 2 Faculty of Chemistry and Chemical Engineering, University of Maribor, Smetanova ulica 17, 2000 Maribor, Slovenia; 3 Faculty of Chemistry and Chemical Technology, University of Ljubljana, Večna pot 113, 1000 Ljubljana, Slovenia; 4 Slovenian NMR Centre, National Institute of Chemistry, Hajdrihova 19, 1000 Ljubljana, Slovenia; 5 EN-FIST Centre of Excellence, Trg Osvobodilne Fronte 13, 1000 Ljubljana, Slovenia; 6 Faculty of Pharmacy, University of Ljubljana, Aškerčeva Cesta 7, 1000 Ljubljana, Slovenia

**Keywords:** glasdegib, salts, X-ray diffraction, ssNMR, stability, solubility

## Abstract

Glasdegib is a recently approved drug for the treatment of acute myeloid leukemia. It is formulated and marketed in monomaleate salt form. In our investigation, we were able to prepare a glasdegib dimaleate form, which could, in theory, exist in double-salt form or as a mixture of salt and co-crystal species. Therefore, the obtained crystals of glasdegib dimaleate were characterized via ^15^N ssNMR and single-crystal X-ray diffraction, which revealed that the obtained glasdegib dimaleate exists in double-salt form. This is a surprising finding based on the p*K*_a_ values for glasdegib and maleic acid. Furthermore, we fully characterized the new dimaleate form using thermal analyses (DSC and TGA) and spectroscopy (IR and Raman). Finally, the physicochemical properties, such as solubility and chemical stability, of both forms were determined and compared.

## 1. Introduction

**Figure 1:**
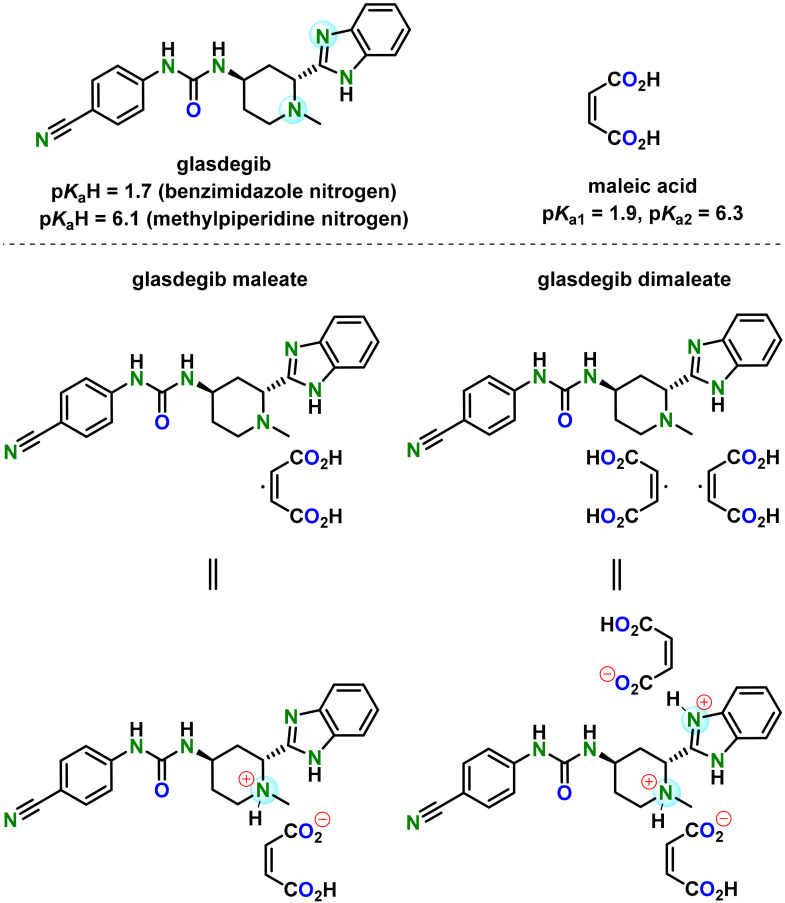
Chemical structure of glasdegib, maleic acid, glasdegib monomaleate and glasdegib dimaleate and their reported p*K*a values [24,25,29].

Acute myeloid leukemia (AML) is a cancer disease that affects the blood and bone marrow. It represents a type of acute leukemia that is most commonly found in adults and can progress rapidly without proper treatment [1]. In recent years, several new therapies for the treatment of AML have appeared. These therapies involve kinase inhibitors (FLT3 inhibitors), IDH1/IDH2 inhibitors, BCL-2 inhibitors, hedgehog inhibitors and others [2,3,4,5,6,7,8,9,10,11,12,13,14,15]. Glasdegib (Figure 1), 1-((2*R*,4*R*)-2-(1*H*-benzo[*d*]imidazol-2-yl)-1-methylpiperidin-4-yl)-3-(4-cyanophenyl)urea (previously also known as PF-04449913), was developed by Pfizer for the treatment of AML [16,17,18,19]. Glasdegib inhibits the hedgehog signaling pathway, known to be associated with a broad range of cancers, via the binding to and inhibition of transmembrane protein Smoothened [17,19,20,21,22,23]. It was approved in November 2018 by the U.S. Food and Drug Administration and in June 2020 by the European Medicines Agency for use, in combination with low-dose cytarabine, as a treatment for newly diagnosed AML in patients aged ≥75 and/or unfit for intensive induction chemotherapy [20,21,22,23].

Glasdegib was developed as a film-coated tablet for oral use which contains the active pharmaceutical ingredient in the form of a monomaleate salt (Figure 1) [24]. Glasdegib was reported to have p*K*_a_ values of 1.7 (benzimidazole nitrogen) and 6.1 (methylpiperidine nitrogen) [24,25], which makes it suitable for the formation of mono-salts with carboxylic acids. Although several salts of glasdegib have been reported in the literature (e.g., maleate, (*S*)-mandelate and dihydrochloride, which forms a hydrate) [17,26], the monomaleate salt has the most favorable properties in terms of chemical stability, thereby forming the lowest levels of (2*S*,4*R*)-epimer under stress conditions (50 °C and 75% relative humidity over 6 weeks) [26]. Furthermore, glasdegib monomaleate is a moderately soluble drug [27] with an aqueous solubility of 1.7 mg/mL [24,25]. Therefore, there is a need for the development of alternative glasdegib salts with better stability and/or solubility.

In our study we were able to isolate a glasdegib dimaleate form upon treatment of glasdegib monomaleate with an additional amount of maleic acid [28]. Based on the p*K*_a_ values of glasdegib [24,25] and maleic acid (p*K*_a1_ = 1.9 and p*K*_a2_ = 6.3) [29], the structure of this novel form with regard to the ionization state was not evident. Thus, in this report, we provide full details on the preparation and characterization of the glasdegib dimaleate form. Interestingly, glasdegib dimaleate proved to be a double-salt form, which is surprising. Furthermore, we compare its properties with its monomaleate analogue.

## 2. Materials and Methods

### 2.1. Materials

For the purpose of this study, glasdegib monomaleate (Taros, Dortmund, Germany) was used.

For the purpose of glasdegib dimaleate synthesis, maleic acid, sodium hydroxide, methanol, ethyl acetate and dichloromethane were purchased from Merck KGaA (Darmstadt, Germany). Hydrochloric acid and isopropanol were obtained from FluoroChem (Derbyshire, UK).

### 2.2. Characterization Methods

#### 2.2.1. Fourier Transform Infrared (FTIR) Measurements

FTIR spectra were collected using a Nicolet™ iS50 FTIR Spectrometer (Thermo Fisher Scientific Inc., Waltham, MA, USA) with a KBr disk.

#### 2.2.2. Raman Measurements

Raman spectra were collected using a Nicolet™ iS50 FTIR Spectrometer (Thermo Fisher Scientific Inc., Waltham, MA, USA) with an iS50 Raman Module.

#### 2.2.3. Differential Scanning Calorimetry (DSC) and Thermogravimetric Analysis (TGA) Measurements

DSC thermograms were acquired using a differential scanning calorimeter DSC 3+ STAR^e^ System instrument (Mettler Toledo, Columbus, OH, USA), and TGA thermograms were collected using a TGA/DSC 1 STAR^e^ System instrument (Mettler Toledo, Polaris Parkway, Columbus, OH, USA). The sample was heated in a 40 microliter aluminum pan with a pierced aluminum lid from 30 to 300 °C at a rate of 10 K/min. Nitrogen (purge rate 200 mL/min) was used as a purge gas.

#### 2.2.4. Powder X-ray Diffraction (PXRD) Measurements

Bulk powder samples of glasdegib base, dihydrochloride, monomaleate and dimaleate were analyzed via powder X-ray diffraction using the PANalytical Empyrean diffractometer (Malvern Panalytical GmbH, Kassel, Germany) equipped with a theta/theta coupled goniometer in transmission geometry, Cu-Kα1, 2 radiation (wavelength 0.15419 nm) with a focusing mirror and a solid state PIXcel1D detector. The patterns (Figures S16, S18 and S20–S23) were recorded at a tube voltage of 45 kV and a tube current of 40 mA, applying a step size of 0.026° 2-Theta with 50 s per step in the angular range of 2° to 40° 2-Theta under ambient conditions. Automatic divergence and antiscatter slits were used to irradiate 10 mm of sample length.

#### 2.2.5. Nuclear Magnetic Resonance (NMR) Spectroscopy

All solutions’ NMR spectra (Figures S27–S30) were recorded at 298 K using a Bruker Avance III 500 MHz spectrometer (Bruker Biospin, Rheinstetten, Germany). The spectrometer was equipped with a 5 mm BBO, Z-gradient probe operating at a ^1^H resonance frequency of 500 MHz and a ^13^C resonance frequency of 125 MHz. Spectra were acquired and processed using Bruker TopSpin software, version 3.1. ^1^H NMR chemical shifts (*δ_H_*) and ^13^C NMR chemical shifts (*δ_C_*) are quoted in parts-per-million (ppm) downfield from tetramethylsilane (TMS), and coupling constants (*J*) are quoted in Hertz (Hz). The abbreviations for NMR data are s (singlet), d (doublet), t (triplet), sept (septet) and m (multiplet).

#### 2.2.6. Solid-State Nuclear Magnetic Resonance (ssNMR) Analysis

ssNMR spectra were acquired using the Agilent Technologies NMR System 600 MHz NMR spectrometer (Varian Inc., Palo Alto, CA, USA) equipped with a 3.2 mm NB dual resonance HX MAS probe. The Larmor frequencies of proton and nitrogen nuclei were 599.39 and 60.75 MHz, respectively. ^1^H NMR chemical shifts are reported relative to external reference adamantane (*δ_H_* 1.85 ppm), which corresponds to the TMS signal at *δ_H_* 0.0 ppm. ^15^N NMR chemical shifts are reported relative to ammonium sulfate (*δ_N_* −355.7 ppm), which corresponds to the nitromethane signal at *δ_N_* 0.0 ppm. Samples were spun at 20,000 (^1^H) and 10,000 Hz (^15^N). A short excitation time of 200 μs was used to transfer polarization, relaxation delays of 1.5–4.75 s and at least 50,000 repetitions.

#### 2.2.7. X-ray Single-Crystal Analysis

Single-crystal X-ray diffraction data for glasdegib monomaleate and glasdegib dimaleate were collected on an Agilent Technologies SuperNova Dual diffractometer (Yarnton, UK) using an Atlas detector with monochromated Cu-Kα radiation (λ = 1.54184 Å) at 150 K. The data were processed using CrysAlis Pro [30]. Structures were solved via the SHELXT program [31] and refined using a full-matrix least-squares procedure based on *F*^2^ with SHELXL [32] using the Olex2 program suite [33]. All non-hydrogen atoms were refined anisotropically. All hydrogen atoms were readily located in difference Fourier maps. Hydrogen atoms bonded to carbon atoms were subsequently treated as riding atoms in geometrically idealized positions with *U*_iso_(H) = *kU*_eq_(C), where *k* = 1.5 for methyl groups—which were permitted to rotate but not to tilt—and 1.2 for all other H atoms. N–H groups were refined by restraining bonding distances, except for H atoms attached to N4 with *U*_iso_(H) = 1.2*U*_eq_(N). Hydrogen atoms involved in the intramolecular O–H⋯O interactions in maleate monoanions were refined freely with *U*_iso_(H) = 1.5*U*_eq_(O). Geometric parameters were calculated using the SHELXL [32] and Platon programs [34]. The crystallographic data are listed in Table 1.

#### 2.2.8. Ultra-High-Performance Liquid Chromatography (UHPLC) Method

The solubility of glasdegib and related substances/degradation products were measured using ultra-high-performance liquid chromatography (UHPLC) with UV detection. For that purpose, an Acquity UPLC system (Waters, Millford, MA, USA) equipped with a quaternary solvent manager (QSM), a sample manager (SM), a temperature-controlled column compartment and a tunable UV (TUV) detector was used. The method conditions were as follows: column: Acquity UPLC CSH C18 (1.7 μm, 100 mm × 2.1 mm); mobile phase A: A = ammonium bicarbonate (pH 9.0; 10 mM) pH-adjusted using ammonia solution (25%); mobile phase B: B = 100% ACN; pump flow: 0.4 mL/min; injection volume: 1 µL; column temperature: 30 °C; autosampler temperature: 10 °C; detection wavelength: 270 nm; needle-wash solvent: ACN-water (1:9, *v*/*v*); purge wash solvent: methanol-water (1:9, *v*/*v*); sample solvent composition: methanol-water (3:7, *v*/*v*); gradient: *t* = 0 min, 15% B; *t* = 0.5 min, 15% B; *t* = 11 min, 40% B; *t* = 11.5 min, 75% B; *t* = 13 min, 75% B; *t* = 13.5 min, 15% B; re-equilibration = 4.5 min; and t_R_ (glasdegib) = 9.0 min (Figure S24). The concentrations of glasdegib salt for solubility measurements and for the determination of related substances/degradation products were 0.2 and 0.5 mg/mL, respectively.

#### 2.2.9. Chiral High-Performance Liquid Chromatography (HPLC) Method

The chiral purity of glasdegib was determined using an Alliance 2695 Separations Module (Waters, Millford, MA, USA) equipped with a quaternary pump, an autosampler, temperature-controlled column compartment and a 2487 Dual Absorbance Detector (Waters, Millford, MA, USA). The method conditions were as follows: column: Chiralpak IC (5 μm, 250 mm × 4.6 mm; Daicel Corp, Tokyo, Japan); mobile phase A: A = 100% heptane; mobile phase B: B = 2-propanol containing 0.1% (*v/v*) diethylamine; pump flow: 1.5 mL/min; injection volume: 10 µL; column temperature: 25 °C; autosampler temperature: 5 °C; detection wavelength: 270 nm; needle-wash solvent: 100% 2-propanol; sample solvent: 100% methanol; gradient: *t* = 0 min, 10% B; *t* = 0.5 min, 10% B; *t* = 20.5 min, 40% B; *t* = 27 min, 60% B; *t* = 28 min, 10% B; re-equilibration = 12 min; t_R_ (glasdegib) = 8.7 min; and t_R_ (2*S*,4*R*-epimer) = 15.8 min (Figure S25). The concentration of glasdegib salt for the determination of chiral purity was 1.0 mg/mL.

#### 2.2.10. Stress Stability Testing

Preliminary stress stability testing was conducted using dynamic vapor sorption apparatus (ProUmid SPSx-1μ Sorption Test System, Ulm, Germany). Samples of glasdegib monomaleate and glasdegib dimaleate were exposed to a temperature of 40 °C and relative humidity of 75% for 3 months. Samples were taken for analysis after 1, 2 and 3 months and analyzed via UHPLC (purity), chiral HPLC (chiral purity) and PXRD (form purity).

#### 2.2.11. Dissolution and Solubility Testing

Dissolution and solubility were evaluated for both glasdegib monomaleate and glasdegib dimaleate. Dissolution was tested at different pH values using the following buffer solutions: pH 1.2 (United States Pharmacopeia 29), pH 4.0 (European Pharmacopoeia 7.0, ref. 4013800), pH 5.5 (European Pharmacopoeia 7.0, ref. 4002000) and pH 7.0 (European Pharmacopoeia 7.0, ref. 4008200) at 37 °C and 300 rpm using an EasyMax 102 reactor system (Mettler Toledo, Greifensee, Switzerland) using 50 mL reactor and a magnetic stirrer. Experiments were performed by adding excess glasdegib salts into the desired buffer solution already set at 37 °C. Samples were taken after stopping the stirring for 5–10 min (decantation) to avoid the contamination of samples with insoluble particles. Samples were then analyzed via UHPLC to measure glasdegib concentration.

### 2.3. Synthesis Methods and Characterization Data

To facilitate the ssNMR characterization of glasdegib dimaleate, standards of glasdegib base and glasdegib dihydrochloride were prepared from glasdegib maleate.

#### 2.3.1. Characterization of Glasdegib Monomaleate

DSC (10 °K/min): 195.4 °C onset, 202.2 °C peak; FTIR (KBr): 3424, 3326, 3297, 3275, 3188, 3042, 2975, 2954, 2912, 2882, 2837, 2787, 2750, 2219, 1690, 1602, 1535, 1499, 1463, 1445, 1411, 1387, 1359, 1329, 1319, 1273, 1261, 1240, 1218, 1174, 1143, 1113, 1096, 1070, 1061, 1030, 1016, 1008, 986, 960, 927, 901, 861, 847, 829, 801, 771, 755, 716, 673, 570, 550, 520, 508, 495, 480, 454 and 420 cm^−1^; Raman: 3118, 3072, 3059, 3029, 3013 2988, 2976, 2955, 2933, 2219, 1691, 1613, 1589, 1535, 1490, 1445, 1432, 1387, 1329, 1320, 1303, 1288, 1273, 1261, 1233, 1208, 1175, 1145, 1113. 1070, 1035, 1023, 1014, 997, 966, 926, 911, 901, 874, 841, 830, 800, 782, 748, 729, 716, 676, 646, 620, 561, 551, 520, 511, 495, 480, 466, 455, 445, 422 and 401 cm^−1^; ^1^H NMR (MeOD, 500 MHz): *δ* 7.68–7.62 (m, 2H), 7.59 (s, 4H), 7.34 (dd, *J* = 6.1, 3.1 Hz, 2H), 6.30 (s, 2H), 4.57 (d, *J* = 4.3 Hz, 1H), 4.27–4.22 (m, 1H), 3.61 (d, *J* = 12.8 Hz, 1H), 3.30–3.23 (m, 1H), 2.67 (s, 3H), 2.48–2.36 (m, 2H), 2.25 (ddt, *J* = 15.1, 11.1, 4.2 Hz, 1H) and 2.08 (dt, *J* = 14.9, 4.5 Hz, 1H) ppm; ^13^C NMR (MeOD, 125 MHz, proton-decoupled): *δ* 170.80, 156.73, 150.78, 145.62, 138.35, 135.92, 134.25, 124.93, 120.16, 119.50, 119.41, 116.29, 105.46, 59.45, 51.82, 43.35, 42.81, 35.46 and 29.08 ppm; PXRD diffractogram (Figure S16) corresponds to the previously published data [26].

#### 2.3.2. Synthesis of Amorphous Glasdegib Base

Ethyl acetate (60 mL) and 0.5 M sodium hydroxide solution (100 mL) were added to glasdegib monomaleate (2.00 g, 4.08 mmol). The suspension was mixed at 25 °C until no solid particles were observed. The phases were separated, and the organic phase was washed two times with 0.5 M sodium hydroxide solution (2 × 45 mL) and another two times with water (2 × 20 mL). Ethyl acetate phase was dried over sodium sulfate. The solids were filtered off, and the filtrate was concentrated on the rotary evaporator until a solid phase was obtained. The amorphous solid was additionally dried at 40 °C under reduced pressure to obtain 1.30 g (65% yield) of amorphous glasdegib base, which was used directly for the preparation of the crystalline glasdegib base.

#### 2.3.3. Synthesis and Characterization of Crystalline Glasdegib Base

The amorphous glasdegib base (200 mg, 0.534 mmol) was dissolved in mixture of ethyl acetate (40 mL) and methanol (0.6 mL) upon heating to 60 °C using an ultrasonic bath. Clear solution was placed into the vial and stirred at room temperature (25 °C) for about 16 h, with a pierced vial cap to enable slow evaporation of part of the solvent. The resulting solid was filtered and dried at 40 °C under reduced pressure to give to give 110 mg (55% yield) of crystalline glasdegib base (Figure S20). DSC (10 °K/min): 235.5 °C onset, 237.2 °C peak; FTIR (KBr): 3356, 3051, 2982, 2947, 284, 2793, 2219, 1708, 1659, 1622, 1593, 1456, 1431, 1418, 1380, 1369, 1328, 1271, 1177, 1146, 1146, 1112, 1073, 1042, 1002, 991, 896, 834, 778, 767, 744, 677, 639, 617, 569, 549, 513, 467 and 424 cm^−1^; Raman: 3353, 3066, 3053, 2982, 2951, 2929, 2897, 2224, 2216, 1709, 1661, 1610, 1592, 1543, 1532, 1455, 1321, 1270, 1230, 1206, 1180, 1147, 1114, 1046, 1028, 1003, 982, 926, 890, 850, 830, 751, 714, 643, 625, 617, 552, 514, 503, 469 and 446 cm^−1^.

#### 2.3.4. Synthesis and Characterization of Glasdegib Dihydrochloride Hydrate

The glasdegib base (100 mg, 0.267 mmol) was dissolved in mixture of ethyl acetate (17 mL) and methanol (3 mL). The suspension was mixed at 50 °C until it became clear. To the solution, 6 N hydrochloric acid in isopropanol (0.1068 mL) was added. After several minutes, a solid phase was observed. The suspension was cooled down to 5 °C, and the solid particles were filtered and dried at 40 °C under reduced pressure to provide 50 mg (42% yield) of crystalline glasdegib dihydrochloride hydrate [17] (Figure S21). DSC (10 °K/min): 159.1 °C onset, 189.4 °C peak and 208.0 °C onset, 210.7 °C peak; FTIR (KBr): 3314, 3100, 3050, 2928, 2853, 2736, 2613, 2224, 1707, 1629, 1595, 1534, 1486, 1471, 1453, 1430, 1414, 1383, 1325, 1288, 1223, 1175, 1151, 1116, 1064, 1023, 989, 968, 930, 896, 876, 836, 759, 750, 668, 638, 620, 546, 518, 495 and 479 cm^−1^; Raman: 3087, 3078, 3053, 3024, 2993, 2982, 2974, 2945, 2934, 2227, 2215, 1715, 1706, 1610, 1573, 1545, 1528, 1491, 1453, 1430, 1388, 1336, 1317, 1306, 1287, 1266, 1242, 1205, 1176, 1155, 1118, 1101, 1064, 1037, 1019, 1001, 968, 930, 896, 878, 843, 831, 800, 745, 714, 704, 644, 620, 561, 544, 520, 509, 495, 474, 446, 417 and 400 cm^−1^.

#### 2.3.5. Synthesis and Characterization of Glasdegib Dimaleate

Glasdegib monomaleate (100 mg, 0.22 mmol) and maleic acid (31 mg, 0.27 mmol) were dissolved in a mixture of dichloromethane (1.9 mL) and methanol (0.1 mL) upon heating to 40 °C and with the aid of sonication in an ultrasonic bath. The obtained clear solution was filtered through a 0.45 μm syringe filter into a fresh vial fitted with a stir bar. Under constant mixing, dichloromethane (12 mL) was added to the solution. After 2 h of stirring, a white suspension was formed, which was further stirred at room temperature for approximately 16 h. The precipitate was isolated via filtration, rinsed with dichloromethane (1 mL) and dried for about 16 h at room temperature and under a reduced pressure of about 50 mbar to obtain 60 mg (49% yield) of crystalline the glasdegib dimaleate form (Figure S18). DSC (10 °K/min): 186.0 °C onset, 190.1 °C peak; FTIR (KBr): 3390, 3326, 3274, 3223, 3183, 3149, 3105, 3047, 2946, 2868, 2704, 2595, 2215, 1699, 1626, 1599, 1536, 1416, 1384, 1355, 1330, 1317, 1291, 1244, 1227, 1176, 1147, 1123, 1096, 1067, 1008, 988, 930, 891, 867, 849, 821, 799, 752, 718, 673, 667, 618, 577, 551, 521, 505, 492, 450 and 427 cm^−1^; Raman: 3192, 3077, 3049, 2990, 2950, 2216, 1699, 1602, 1562, 1536, 1454, 1381, 1347, 1318, 1292, 1271, 1209, 1176, 1146, 1119, 1110, 1037, 1018, 999, 964, 932, 894, 876, 864, 843, 830. 799, 782, 745, 729, 715, 618 and 486 cm^−1^; ^1^H NMR (MeOD, 500 MHz): *δ* 7.70–7.63 (m, 2H), 7.59 (s, 4H), 7.40–7.32 (m, 2H), 6.31 (s, 4H), 4.71 (dd, *J* = 8.9, 4.9 Hz, 1H), 4.26 (t, *J* = 4.6 Hz, 1H), 3.70 (d, *J* = 12.9 Hz, 1H), 3.37 (ddd, *J* = 14.1, 11.3, 3.3 Hz, 1H), 2.74 (s, 3H), 2.52–2.41 (m, 2H), 2.28 (ddt, *J* = 15.0, 11.0, 4.2 Hz, 1H) and 2.11 (dt, *J* = 15.2, 4.6 Hz, 1H) ppm; ^13^C NMR (MeOD, 125 MHz, proton-decoupled): *δ* 169.93, 156.75, 149.89, 145.59, 138.31, 134.24, 133.74, 125.11, 120.16, 119.53, 119.44, 116.36, 105.47, 59.31, 51.86, 43.22, 42.56, 35.16 and 28.83 ppm; PXRD (Cu-Kα): 5.5, 7.5, 9.2, 10.3, 11.0, 11.3, 11.9, 13.8, 14.7, 15.4, 17.0, 17.3, 18.0, 18.6, 19.3, 20.3, 20.8, 22.0, 22.8, 24.6, 25.4, 25.6, 26.0, 26.6, 27.3, 27.9, 29.2 and 29.8° 2θ.

## 3. Results

### 3.1. Synthesis of Glasdegib Dimaleate

**Figure 2:**
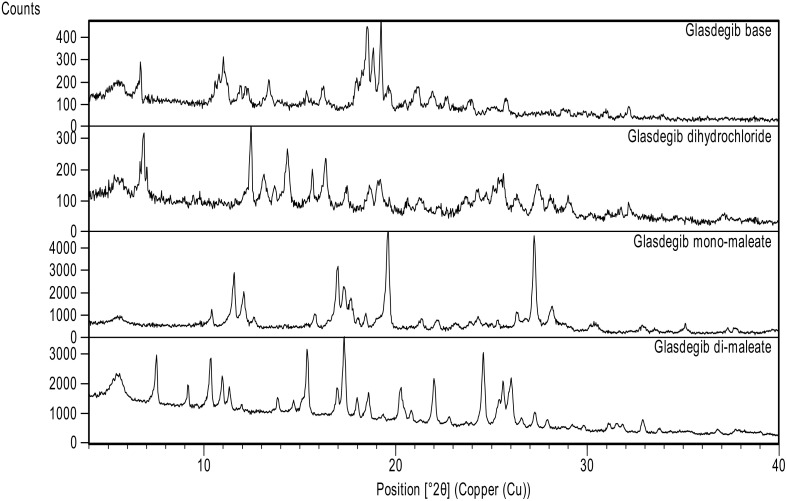
Powder X-ray diffraction patterns of glasdegib derivatives: glasdegib base, glasdegib dihydrochloride, glasdegib monomaleate and glasdegib dimaleate.

Initially we conducted an extensive screening of the solid form in order to identify potential novel forms of glasdegib. In the majority of cases, no new solid form was detected. However, when we crystalized glasdegib monomaleate from dichloromethane, we observed that the substance was only partially soluble. Furthermore, after the removal of undissolved material via filtration, a new solid was crystallized from a clear dichloromethane solution. The analyses of the solid via PXRD (Figure 2, and Figures S16, S18, S20 and S21) and DSC (Figures S1–S4) suggested that a new form was obtained. Moreover, the solution ^1^H NMR analysis of the obtained solid indicated that excess maleic acid was present in a novel form. Therefore, subsequent targeted crystallization experiments using glasdegib monomaleate in the presence of additional quantities of maleic acid provided a new form, which was determined to be glasdegib dimaleate based on the solution ^1^H NMR analysis (Figures S27 and S29). In order to improve the process feasibility, due to the low solubility of glasdegib monomaleate in dichloromethane, we conducted subsequent crystallization experiments from the mixture of dichloromethane and methanol, which provided consistently pure glasdegib dimaleate.

### 3.2. Characterization of Glasdegib Dimaleate

#### 3.2.1. PXRD Analysis

As presented in Figure 2 and Figure S18, glasdegib dimaleate had notably different PXRD signal pattern in the PXRD diffractogram compared to the glasdegib monomaleate, glasdegib base or glasdegib dihydrochloride. The PXRD pattern of glasdegib dimaleate contained several characteristic signals at 2-Theta of 7.5°, 15.4°, 20.3°, 20.8°, 22.0°, 24.6°, 25.4°, 25.6° and 26.0°, which were not observed in the glasdegib monomaleate, glasdegib base or glasdegib dihydrochloride. To obtain a more complete picture on the characteristics of this novel form, we performed additional characterization using IR, Raman, DSC, TGA ssNMR and single-crystal X-ray analysis, which is described below.

#### 3.2.2. Infrared Spectral Analysis

**Figure 3:**
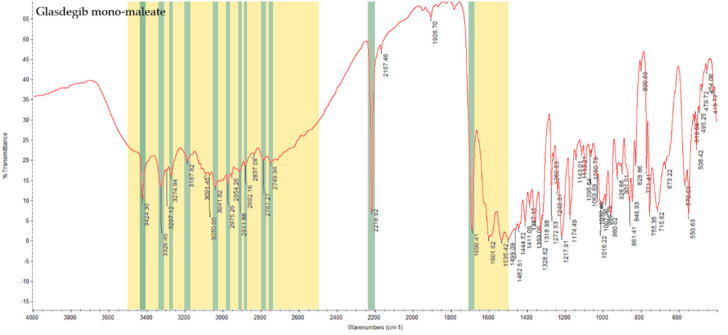
IR spectrum of glasdegib monomaleate with highlighted diagnostic regions and bands.

**Figure 4:**
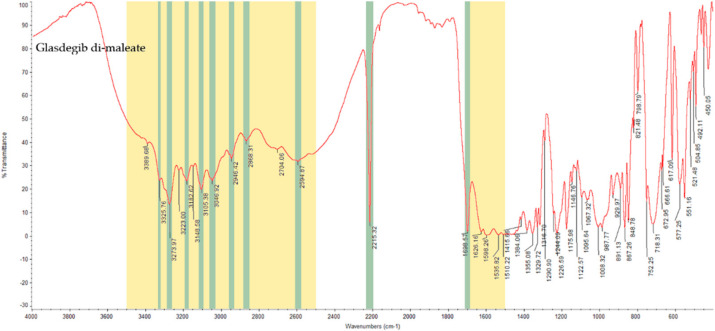
IR spectrum of glasdegib dimaleate with highlighted diagnostic regions and bands.

In the IR spectrum of glasdegib monomaleate (Figure 3) and glasdegib dimaleate (Figure 4), the region between 3500 and 2500 cm^−1^ is populated by overlapping N–H, O–H, *sp^2^* C–H (benzene rings) and *sp^3^* C–H (CH_2_ and CH_3_ groups) stretching vibrations. In the case of the monomaleate form, distinct bands are observed at 3424, 3326, 3275, 3188, 3042, 2975, 2912, 2882, 2787 and 2750 cm^−1^ (Figure 3), while in the case of the dimaleate form, distinct bands are observed at 3326, 3274, 3183, 3105, 3047, 2946, 2868 and 2595 cm^−1^ (Figure 4). The most diagnostic band in the IR spectra is associated with stretching of the nitrile (C≡N) group, which is located at 2219 cm^−1^ for the monomaleate form (Figure 3) and at 2215 cm^−1^ for the dimaleate form (Figure 4). The carbonyl (C=O), alkene (C=C) and aromatic (C=C) stretching vibration regions (ca. 1700–1500 cm^−1^) are densely populated with bands for the *cis*-alkene group, aromatic double bonds, the urea carbonyl group, the carboxylic acid carbonyl group and the carboxylate anion carbonyl group. In this region the most diagnostic band is located at 1690 cm^−1^ for the monomaleate form (Figure 3) and at 1699 cm^−1^ for the dimaleate form (Figure 4). Similarly, the fingerprint regions or IR spectra for both forms are crowded with a large number of similar absorption bands.

#### 3.2.3. Raman Analysis

**Figure 5:**
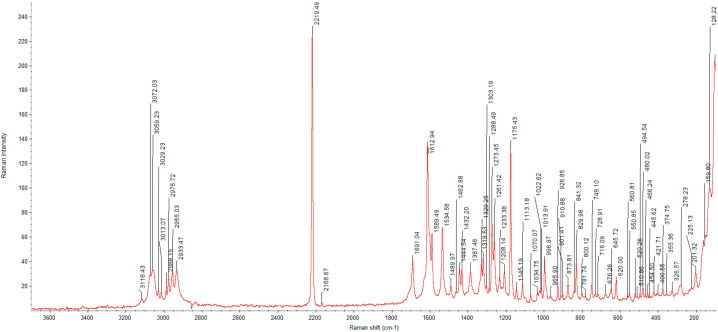
Raman spectrum of glasdegib monomaleate.

**Figure 6:**
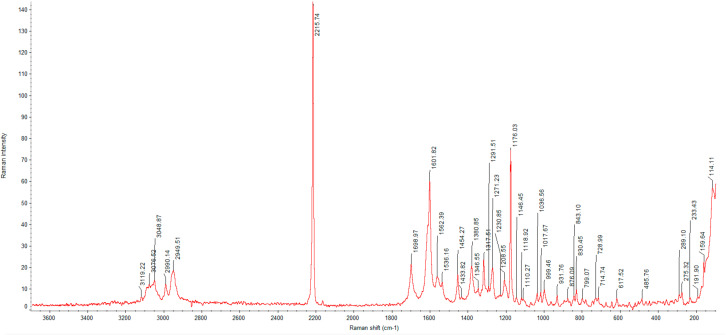
Raman spectrum of glasdegib dimaleate.

Due to the congested IR spectrum with a large number of overlapping bands in the fingerprint region, we decided to also characterize the glasdegib monomaleate and dimaleate forms using Raman spectroscopy, which should enable better distinction of both forms. The Raman spectrum of glasdegib monomaleate (Figure 5) and glasdegib dimaleate (Figure 6) display CH stretching of unsaturated carbons in the region above 3000 cm^−1^, while CH stretching of saturated carbons popthe ulates the region from 3000 to 2933 cm^−1^ for monomaleate form, and from 3000 to 2950 cm^−1^ for the dimaleate form. The most diagnostic bands (C≡N stretch of a nitrile bond) in the Raman spectra are located at 2219 cm^−1^ for the monomaleate form (Figure 5) and at 2216 cm^−1^ for the dimaleate form (Figure 6). Furthermore, a distinct characteristic band is also observed for the carbonyl group (C=O) at 1691 cm^−1^ for the monomaleate form (Figure 5) and at 1699 cm^−1^ for the dimaleate form (Figure 6). In addition, very strong aryl C=C stretches at 1613 cm^−1^ for the monomaleate form (Figure 5) and 1602 cm^−1^ for the dimaleate form (Figure 6) are observed, and are also detected in the glasdegib base (Figure S13).

#### 3.2.4. DSC and TGA Analysis

**Figure 7:**
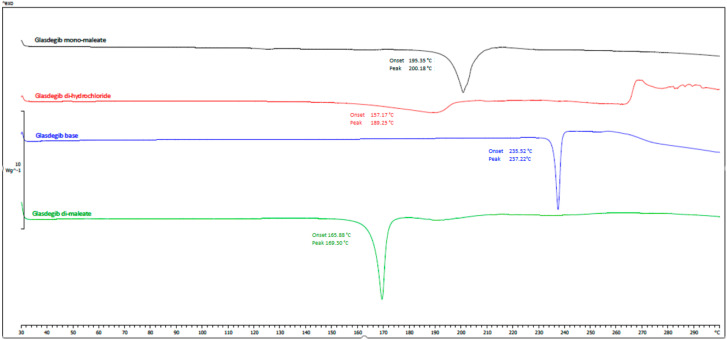
DSC thermograms of glasdegib derivatives. Black: glasdegib monomaleate, red: glasdegib dihydrochloride hydrate; blue: glasdegib base; and green: glasdegib dimaleate.

The thermal behaviors of the obtained glasdegib dimaleate, glasdegib monomaleate, glasdegib dihydrochloride hydrate [17] and glasdegib base are shown in Figure 7 and Figures S1–S4. It can be observed that the new dimaleate form exhibits distinct thermal behavior compared to the other glasdegib derivatives (Figure 7).

In the DSC thermogram of glasdegib monomaleate (Figure 7, black line and Figure S1) two endothermic transitions were observed. The first transition at 118.5 °C (onset) and 124.8 °C (peak) is associated with a 2.3% mass loss, as observed in the TGA thermogram above 100 °C (Figure S1), which is probably associated with the partial desolvation of the residual solvent. The second broader peak transition at 195.4 °C (onset) and 200.2 °C (peak) is associated with the melting of the monomaleate form. After both endothermic phenomena, an exothermic transition associated with decomposition was not observed up to 300 °C.

Glasdegib dihydrochloride hydrate (Figure 7, red line and Figure S4) exhibited more complex thermal behavior, where a very broad endothermic peak was observed at 159.1 °C (onset) and 189.4 °C (peak). In this endothermic transition, the TGA thermogram (Figure S4) shows that 10.86% of the mass is lost until 206 °C, which probably corresponds to a loss of one hydrochloric acid and one water (calculated mass loss 11.7%). Subsequently, another endothermic transition is observed at 208.0 °C (onset) and 210.7 °C (peak), which probably corresponds to the melting of the formed hydrochloride form. In the TGA thermogram (Figure S4) it can be observed that additional mass loss starts at temperatures above 215 °C and continues up to the final measured temperature of 300 °C. In this temperature range, the formation of the glasdegib base (see paragraph below) can be observed in the DSC thermogram, which melts at 235.7 °C (onset) and 237.8 °C (peak). Interestingly, at temperatures between 265 and 270 °C, an exothermic transition can be observed, which is not observed in other glasdegib derivatives and is probably associated with decomposition.

The glasdegib base (Figure 7, blue line and Figure S3) has a simple thermogram with a single melting peak at 235.2 °C (onset) and 237.2 °C (peak) and no exothermal transition up to 300 °C. The mass loss for the glasdegib base was observed in the TGA thermogram (Figure S3) above 230 °C and continued until the final measured temperature of 300 °C.

The DSC curve of glasdegib dimaleate (Figure 7, green line and Figure S2) shows no thermal events until the first endothermic peak, with an onset temperature of 165.9 °C and a peak temperature of about 169.3 °C; this indicates the good thermal stability of the glasdegib dimaleate form. Furthermore, no exothermic phenomena are observed in the DSC thermogram up to 300 °C. The TGA thermogram (Figure S2) shows that mass loss for glasdegib dimaleate starts at temperatures above 110 °C.

#### 3.2.5. Solid-State Nuclear Magnetic Resonance Analysis

In order to determine the true ionization state of glasdegib dimaleate, we first conducted an ssNMR study, which involved ^1^H ssNMR as well as ^15^N ssNMR measurements using glasdegib monomaleate, glasdegib dihydrochloride and glasdegib bases as key standards for the ionized and unionized state of glasdegib. The glasdegib base presents a reference substance for non-protonated species of glasdegib derivatives, whereas glasdegib dihydrochloride serves as a reference for the protonated double-salt form.

The ssNMR characterization of different glasdegib forms included ^1^H echo MAS (magic-angle spinning) ssNMR spectra, which showed proton signals between *δ_H_* 14 and 16 ppm for glasdegib (*δ_H_* 14.6 ppm) as well as dihydrochloride (*δ_H_* 15.8 ppm) that are most likely involved in intermolecular hydrogen bonds (Figure S26). The signal of dihydrochloride salt appears broader, which could be attributed to the fact that multiple molecules are present per asymmetric unit, as also observed in ^15^N ssNMR spectra (vide infra). Glasdegib monomaleate and glasdegib dimaleate showed two sets of signals each: one at *δ_H_* 14–16 ppm and the other at *δ_H_* 20 ppm (Figure S26). The latter suggests the formation of additional hydrogen bonds for monomaleate and dimaleate species. A ^1^H ssNMR chemical shift of such high value is typical for strong hydrogen-bond interactions.

**Figure 8:**
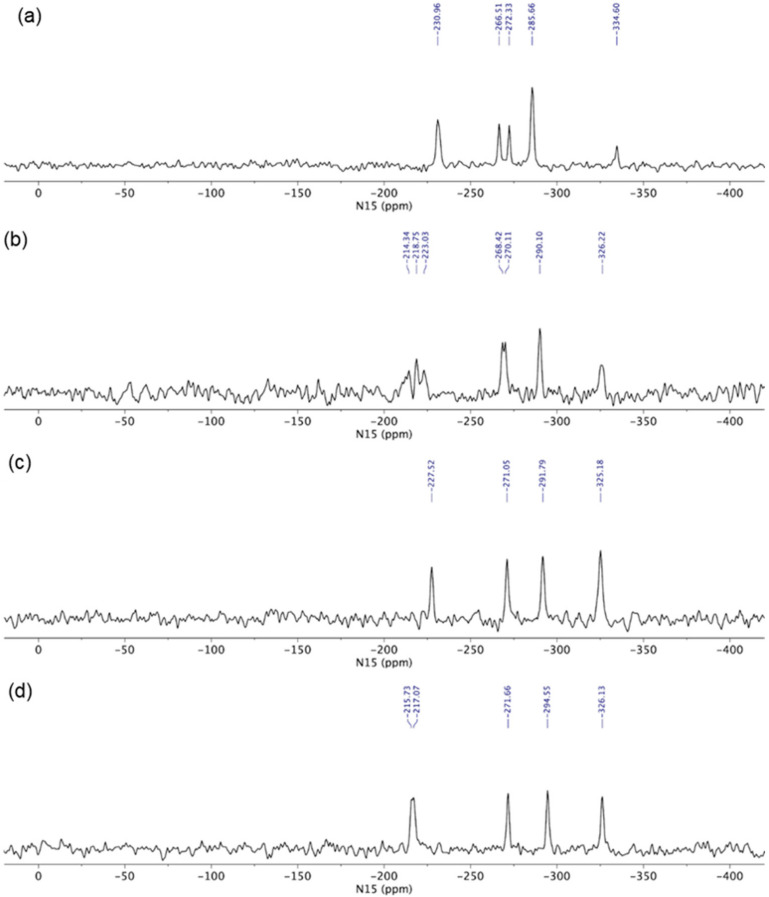
^15^N CP MAS ssNMR (^15^N cross-polarization magic-angle spinning solid-state nuclear magnetic resonance) spectra of (**a**) glasdegib base, (**b**) glasdegib dihydrochloride, (**c**) glasdegib monomaleate and (**d**) glasdegib dimaleate.

The glasdegib base showed four groups of signals in the ^15^N CP-MAS ssNMR spectrum (Figure 8a). The lowest NMR chemical shift was assigned to methylpiperidine nitrogen (*δ_N_* −334.6 ppm). The intense signal at *δ_N_* −285.7 ppm was attributed to both urea nitrogen atoms, followed by two benzimidazole nitrogen atoms in the region between *δ_N_* −266.5 and −272.3 ppm (pyrrole-like NH) and at *δ_N_* −231.0 ppm (pyridine-like N). Similarly, four groups of NMR signals were observed for glasdegib dihydrochloride (Figure 8b). The lowest NMR chemical shift at *δ_N_* −326.2 ppm was assigned to methylpiperidine nitrogen. The signal at *δ_N_* −290.1 ppm was attributed to both urea nitrogen atoms, followed by two benzimidazole nitrogen atoms in the regions between *δ_N_* −268 and −270 ppm (pyrrole-like NH) and between *δ_N_* −210 and −223 ppm (pyridine-like N). Note that some of the NMR signals in the ^15^N CP-MAS spectra of glasdegib dihydrochloride are doubled, which suggests that more than one molecule is present per asymmetric unit (Figure 8b).

Glasdegib dihydrochloride showed a higher intensity of methylpiperidine nitrogen compared to the glasdegib base. Furthermore, the protonation of the methylpiperidine nitrogen atom showed an NMR chemical shift change of ca. 10 ppm (Figure 8a,b).

The ^15^N ssNMR spectra of the glasdegib monomaleate and glasdegib dimaleate species were compared to the glasdegib base and dihydrochloride forms (Figure 8). The methylpiperidine nitrogen atoms of both glasdegib monomaleate and glasdegib dimaleate showed ^15^N chemical shifts similar to glasdegib dihydrochloride, which suggests the protonation of methylpiperidine nitrogen. A chemical shift of the methylpiperidine nitrogen atom was observed at *δ_N_* −325.2 ppm for glasdegib monomaleate species (Figure 8c) and at *δ_N_* −326.1 ppm for glasdegib dimaleate species (Figure 8d). In addition, a small chemical-shift difference was observed for pyridine-like the benzimidazole nitrogen atom when comparing glasdegib monomaleate and glasdegib dimaleate, where dimaleate species showed NMR chemical shifts similar to glasdegib dihydrochloride (compare ^15^N NMR signals at ca. *δ_N_* −216 ppm).

**Figure 9:**
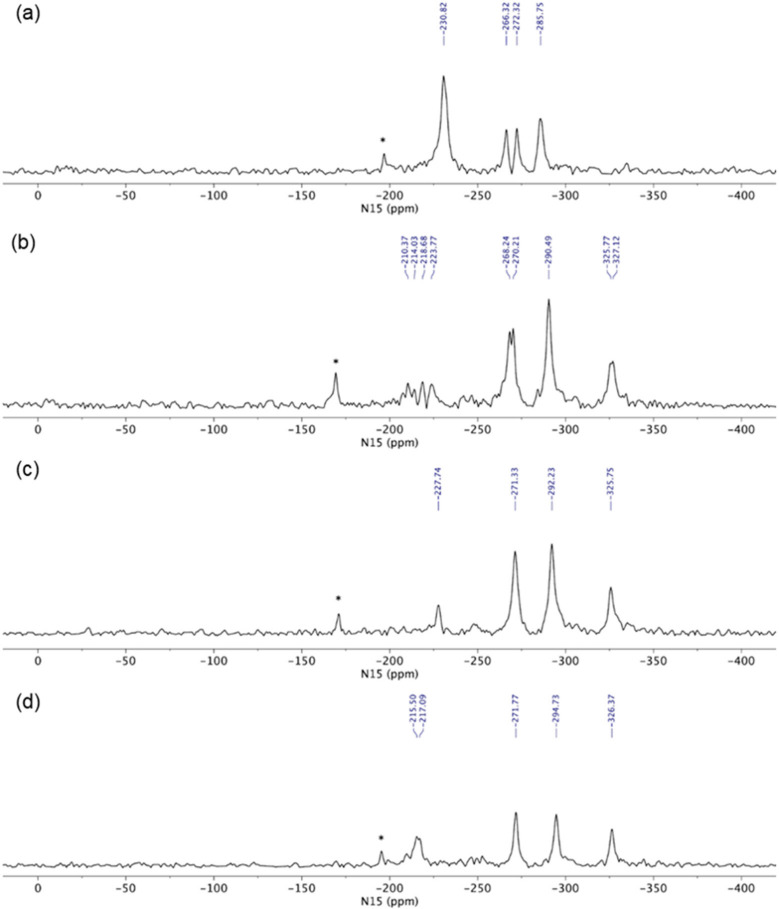
^15^N LG-CP MAS ssNMR (^15^N Lee–Goldburg cross-polarization magic-angle spinning solid-state nuclear magnetic resonance) spectra of (**a**) glasdegib base, (**b**) glasdegib dihydrochloride, (**c**) glasdegib monomaleate and (**d**) glasdegib dimaleate. Note that signals marked with * were attributed to artefacts that correspond to transmitter-frequency offset.

The ^15^N LG-CP MAS ssNMR spectra [35,36,37] were recorded for each glasdegib form, where a short excitation time (200 μs) was used to transfer polarization from the ^1^H to ^15^N nuclei. The comparison with the CP MAS spectra of glasdegib derivatives allowed enabled the detection of protonated and unprotonated species (Figure 9). The ^15^N ssNMR chemical shift of the glasdegib base is consistent with the non-protonated form, as no signal could be observed for the methylpiperidine nitrogen atom at ca. *δ_N_* −335 ppm (Figure 9a). The NMR signals of other nitrogen atoms (urea and benzimidazole nitrogens) were observed at the same chemical shifts as in the ^15^N CP-MAS spectra (compare Figure 8a and Figure 9a).

The ^15^N LG-CP MAS ssNMR spectra confirmed that both monomaleate and dimaleate species correspond to protonated forms of glasdegib (Figure 9c and Figure 9d, respectively). Strong ^15^N NMR signals were observed for methylpiperidine nitrogen atoms in the case of glasdegib monomaleate at *δ_N_* −325.8 ppm (Figure 9c) as well as in the case of glasdegib dimaleate at *δ_N_* −326.4 ppm (Figure 9d). Furthermore, the pyridine-like benzimidazole nitrogen atom of glasdegib monomaleate species showed a negligible shift when compared to the glasdegib base (*δ_N_* −227.7 ppm; Δ*δ_N_* 3 ppm), which suggests that glasdegib monomaleate exists in salt form with its primary protonation site on the methylpiperidine nitrogen atom. Glasdegib dimaleate showed an identical deshielded methylpiperidine nitrogen atom, and interestingly, a remarkable shift in the pyridine-like benzimidazole nitrogen atom (*δ_N_* −216 ppm; Δ*δ_N_* 15 ppm) with respect to the glasdegib base.

In essence, the salt/co-crystal properties of glasdegib monomaleate were assessed by comparing its ^15^N chemical shifts to the glasdegib base and dihydrochloride species. The methylpiperidine nitrogen atoms of glasdegib monomaleate exhibit *δ_N_* −325.8 ppm, which is consistent with the downfield shift of ca. 10 ppm, as observed in the case of glasdegib dihydrochloride. Additionally, the pyridine-like benzimidazole nitrogen atom of the glasdegib monomaleate species showed a negligible shift when compared to the glasdegib base (Δ*δ_N_* 3 ppm), which suggests that glasdegib monomaleate exists in salt form with its primary protonation site on the methylpiperidine nitrogen atom. Glasdegib dimaleate showed and identical deshielded methylpiperidine nitrogen atom (*δ_N_* −326.1 ppm), and moreover, a remarkable shift in the pyridine-like benzimidazole nitrogen atom (Δ*δ_N_* 15 ppm). The latter implies that despite the low Δp*K*a value for the benzimidazole nitrogen/maleic acid pair, the obtained glasdegib dimaleate exists in double-salt form.

#### 3.2.6. X-ray Single-Crystal Determination

**Figure 10:**
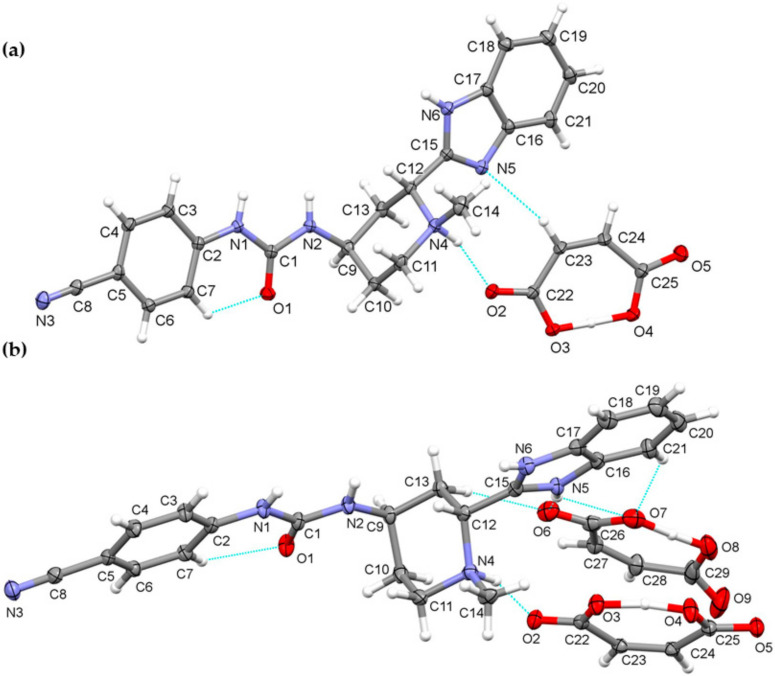
Thermal ellipsoid figures of (**a**) glasdegib monomaleate and (**b**) glasdegib dimaleate drawn at the 30% probability level. The asymmetric unit of glasdegib monomaleate contains one protonated glasdegib molecule and one maleate monoanion. The asymmetric unit of glasdegib dimaleate contains one double-protonated glasdegib molecule and two maleate monoanions. Hydrogen bonds are represented by dashed blue lines.

We were able to obtain crystals of glasdegib monomaleate and glasdegib dimaleate suitable for the X-ray structural analysis (Figure 10a,b). The crystallographic data are listed in Table 1. In both crystal structures, partially deprotonated maleic acid is present in the form of maleate monoanions with a strong intramolecular O–H⋯O hydrogen bond (2.386(4)–2.403(2) Å). The C–O distances are within the ranges expected for maleate monoanion.

Compound glasdegib monomaleate crystalizes in monoclinic space group *P*2_1_ with one protonated glasdegib molecule and one maleate monoanion in an asymmetric unit (Figure 10a).

**Figure 11:**
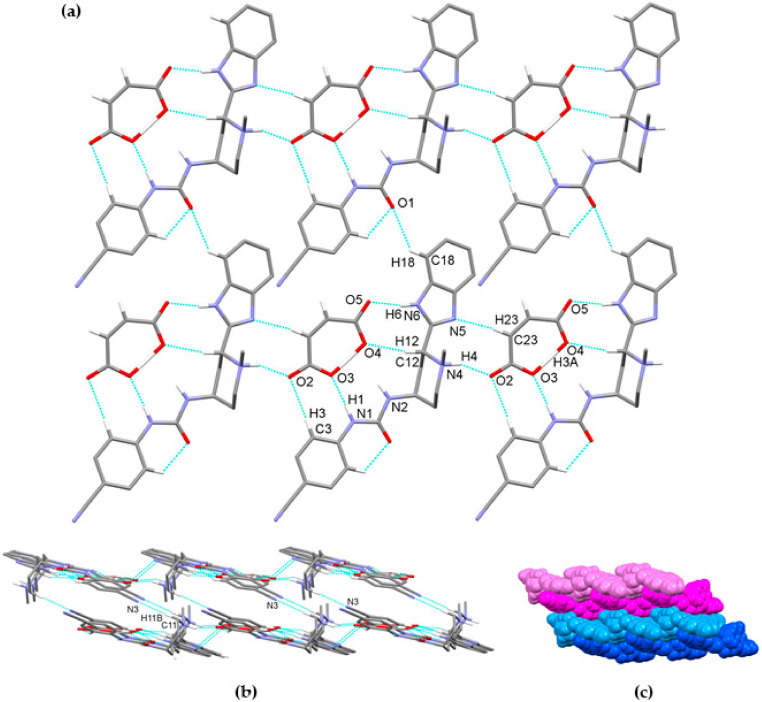
Hydrogen-bond architecture in the glasdegib monomaleate: (**a**) layer formation along *bc* plane; (**b**) formation of double layers via C11–H11b⋯N3 interactions; (**c**) packing of double layers along *a* axis (arbitrary colors; one double layer is presented by light and dark blue and second double layer is represented by light and dark magenta). Hydrogen bonds are represented by dashed blue lines. Hydrogen atoms not involved in the motif shown have been omitted for clarity.

The glasdegib molecule is protonated on the methylpiperidine N4 nitrogen atom. In glasdegib monomaleate, a hydrogen-bonded chain is formed along the *c* axis through a series of interactions between a protonated glasdegib molecule and a maleate monoanion (Figure 11). The methylpiperidine NH group of glasdegib is connected to maleate via N4–H4⋯O2 hydrogen bonding and supported by a C23–H23⋯N5 interaction between maleate and a benzimidazole moiety, forming an R^2^_2_(9) ring motif [38]. Furthermore, glasdegib interacts with the adjacent maleate monoanion through N1–H1⋯O3^i^ and N6–H6a⋯O5^i^ interactions with urea and benzimidazole moieties, respectively, supported by C3–H3⋯O2^i^ and C12–H12⋯O4^i^ bonding and forming one R^2^_2_(12) and two R^2^_2_(8) ring motifs (symmetry code: (i) *x*, *y*, 1 + *z*) (Table 2). The chains are connected into layers along the *bc* plane through C18–H18⋯O1^iii^ interactions between the benzimidazole moiety and the urea oxygen atom (symmetry code: (iii) *x*, 1 + *y*, *z*) (Figure 11a). Double layers are formed through C11–H11b⋯N3^ii^ interaction, connecting the methylpiperidine moiety of one glasdegib molecule with the cyano-group of the adjacent glasdegib molecule (symmetry code: (ii) 1 − *x*, ½ + *y*, 2 − *z*) (Figure 11b,c).

**Figure 12:**
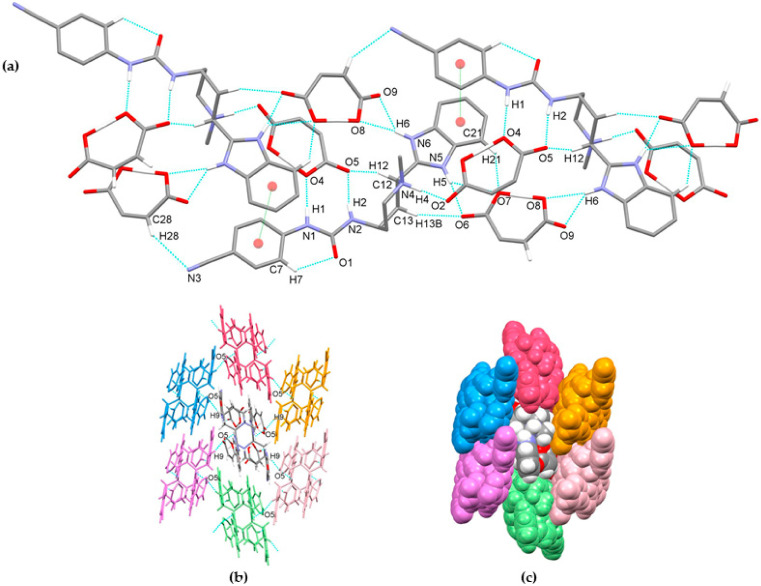
Hydrogen-bond architecture in the glasdegib dimaleate: (**a**) chain formation along *c* axis; (**b**) packing of chains along *ab* plane via C9–H9⋯O5 interactions; (**c**) space-filled presentation of packing of chains (arbitrary colors). Hydrogen bonds are represented by dashed blue lines and π⋯π interactions by dashed green lines. Hydrogen atoms not involved in the motif shown have been omitted for clarity.

Compound glasdegib dimaleate crystalizes in orthorhombic space group *P*2_1_2_1_2_1_ with one double-protonated glasdegib molecule and two maleate monoanions in an asymmetric unit (Figure 10b). The glasdegib molecule is protonated on the methylpiperidine N4 nitrogen atom as well as on the benzimidazole N5 nitrogen atom. A small structural difference between the monoprotonated vs. diprotonated glasdegib molecule is also evident due to the C15–N5 and C15–N6 distances being 1.315(3) and 1.359(3) Å in glasdegib monomaleate vs. 1.331(4) and 1.328(4) Å in glasdegib dimaleate, respectively. In glasdegib dimaleate, a hydrogen-bonded chain is formed along the *c* axis through a series of interactions between the double-protonated glasdegib molecule and two maleate monoanions (Figure 12). The methylpiperidine moiety of the glasdegib dication is connected to one maleate anion via N4–H4⋯O2 hydrogen bonding, and this maleate anion is further connected to the adjacent glasdegib dication through N1–H1⋯O4^i^ and N2–H2⋯O5^i^ interactions with the urea moiety supported by a C12–H12⋯O5^i^ interaction with the methylpiperidine moiety, forming R^2^_2_(8) and R^1^_2_(7) ring motifs (symmetry code: (i) ½ − *x*, 1 − *y*, ½ + *z*) (Figure 12a, Table 2). The benzimidazole moiety interacts with one maleate monoanion through bifurcated N5–H5⋯O6 and N5–H5⋯O7 hydrogen bonding, supported by C13–H13b⋯O6 and C21–H21⋯O7 interactions; thus, each oxygen atom (O6 and O7) is an acceptor of two hydrogen bonds, and R^1^_2_(7), R^1^_2_(6) and R^2^_1_(4) ring motifs are formed. Furthermore, the benzimidazole moiety also interacts with the adjacent maleate monoanion through bifurcated N6–H6⋯O8^i^ and N6–H6⋯O9^i^ hydrogen bonding, forming an R^2^_1_(4) ring motif. This maleate monoanion also supports the formation of the chain through a C28–H28⋯N3^iii^ interaction with the cyano-group of the adjacent glasdegib dication (symmetry code: (iii) *x*, *y*, −1 + *z*). Chain formation is also further supported by almost-parallel π⋯π interactions between the phenyl rings of the cyanophenyl and benzimidazole moieties of two adjacent molecules, with a centroid-to-centroid distance of 3.9040(18) Å and ring slippage of 1.413 Å. A supramolecular structure is achieved, connecting the chains via C9–H9⋯O5^ii^ interactions (symmetry code: (ii) 1 − *x*, −½ + *y*, ½ − *z*) (Figure 12b,c).

**Figure 13:**
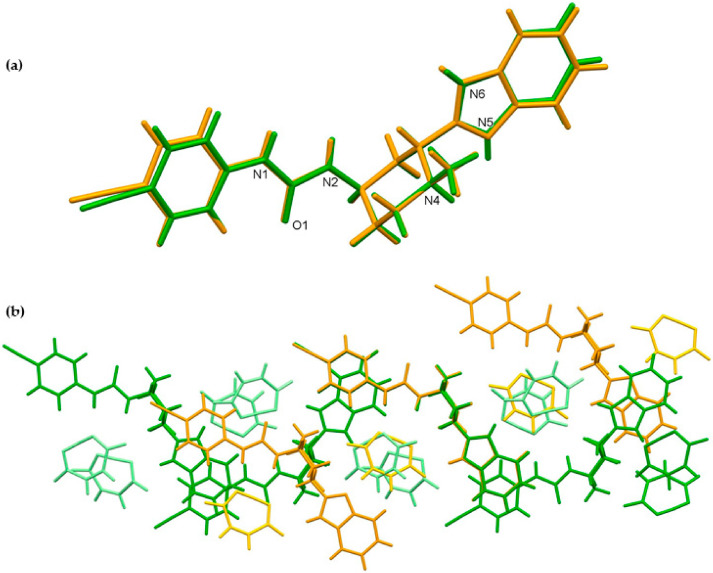
Superposition showing the differences in the conformation of glasdegib monomaleate (orange) and glasdegib dimaleate (green) of (**a**) glasdegib molecule and (**b**) chains along *c* axes, formed primarily by N–H⋯O interactions. Maleate monoanions are presented in light orange and light green colors.

The crystal structures of glasdegib monomaleate and glasdegib dimaleate differ primarily in their glasdegib:maleate monoanion ratios and in a charge on the glasdegib species. In glasdegib monomaleate, each protonated glasdegib molecule is hydrogen-bonded to two adjacent maleate monoanions, forming a chain along the *c* axis, primarily through N–H⋯O interactions. In glasdegib dimaleate, each double-protonated glasdegib molecule is hydrogen-bonded to four adjacent maleate monoanions, forming a chain along the *c* axis, also primarily through N–H⋯O interactions. Monoprotonated and diprotonated glasdegib species differ in their number of potential NH hydrogen-bond donating sites as well as in their N hydrogen-bond acceptor sites. Different hydrogen-bonding patterns can be observed due to the different behavior of urea NH groups. In glasdegib monomaleate, only one urea NH group is involved in hydrogen bonding, while both urea NH groups are hydrogen-bond donors in glasdegib dimaleate. The urea oxygen atom is in both crystal structures involved only in intramolecular hydrogen bonding. In both structures, the methylpiperidine N atom is protonated and is involved in strong N–H⋯O interactions with maleate monoanion. The main difference regarding hydrogen-bond donor/acceptor sites lies in the benzimidazole group. Nitrogen atom N5 is protonated only in glasdegib dimaleate; thus, the benzimidazole moiety possesses two NH groups as a hydrogen-bond donor. Contrarily, in glasdegib monomaleate, benzimidazole possesses only one NH group acting as hydrogen-bond donor and one N atom acting as hydrogen-bond acceptor. In glasdegib monomaleate, four NH groups form three N–H⋯O interactions with maleate monoanion, while in glasdegib dimaleate, five NH groups form seven N–H⋯O interactions, with four being bifurcated. A higher number of hydrogen-bonding interactions in the dimaleate form potentially explains the slightly higher stability of this form. Even though in glasdegib dimaleate, two monoanionic maleate ions are present and the glasdegib is double-protonated, as well as possessing a higher number of hydrogen-donating sites, there is an almost negligible difference in the conformation of mono- vs. diprotonated glasdegib species (Figure 13a). Although their conformations are similar, their intermolecular interactions still lead to different packing arrangements; this is already shown by the fact that glasdegib monomaleate and glasdegib dimaleate crystallize in monoclinic and orthorhombic space groups, respectively. In glasdegib monomaleate, the protonated glasdegib molecules are arranged in a head-to-head fashion, while in glasdegib dimaleate, the double-protonated glasdegib molecules are arranged in a head-to-tail fashion (Figure 13b).

#### 3.2.7. Stability Testing

To gain initial insights into the stability of glasdegib dimaleate, we conducted preliminary comparative stress testing with glasdegib monomaleate in a 3-month study. The stress stability testing of both glasdegib monomaleate and glasdegib dimaleate was conducted under storage conditions of 40 °C and 75% RH (open dish). Both samples were analyzed for purity and chiral purity using liquid chromatography methods at four time points: initially (t = 0), and after each month and was completed after 3 months (Table 3). In addition, solid-form purity was also checked wiusingth PXRD.

The testing results of both glasdegib monomaleate and glasdegib dimaleate indicated that both forms are stable in terms of chiral purity, indicating that the previously reported epimerization of the dihydrochloride form to (2*S*,4*R*)-epimer [26] does not take place in either maleate form in a solid state. Interestingly, the study showed that the monomaleate form is chemically slightly less stable, as evidenced by the analysis of UHPLC purity, where a slightly higher rise in degradation products was observed for glasdegib monomaleate (purity dropped from a 99.86 area% at the beginning of the testing to a 99.72 area% after 3 months; Δ(Σ impurities) = 0.14%) compared to glasdegib dimaleate (purity dropped from 99.98 area% at the beginning of the testing to 99.90 area% after 3 months; Δ(Σ impurities) = 0.08%). The results obtained using the PXRD measurements indicated that glasdegib dimaleate is fully stable for at least 2 months at 40 °C and 75% RH (Figure S22), while in the case of glasdegib monomaleate, traces of glasdegib dimaleate were already observed after 1 month (Figure S23).

#### 3.2.8. Solubility Testing

The dissolution and solubility of glasdegib monomaleate and glasdegib dimaleate were evaluated at a pH of 1.2, 4.0, 5.5 and 7.0 using the United States Pharmacopeia and European Pharmacopoeia buffers.

**Figure 14:**
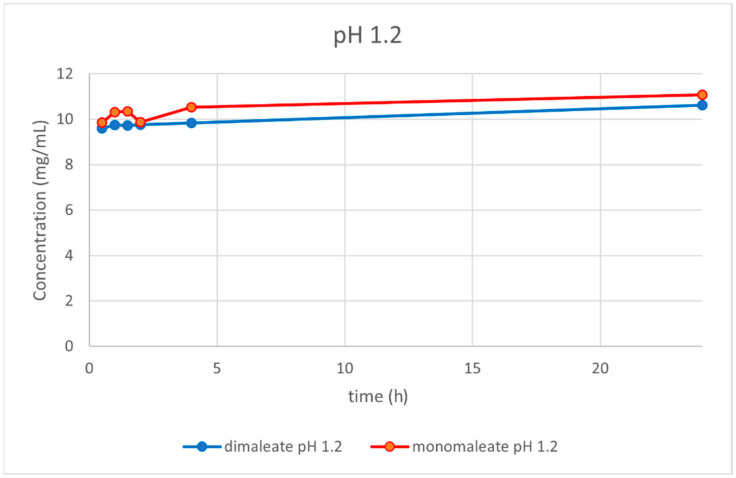
Dissolution testing of glasdegib monomaleate and glasdegib dimaleate at pH = 1.2.

At pH = 1.2 (Figure 14), similar dissolution behavior was observed for both the mono and dimaleate form, with solubility slightly rising over the time. After 24 h, glasdegib monomaleate had slightly higher solubility (11.08 mg/mL) compared to the glasdegib dimaleate (10.62 mg/mL).

**Figure 15:**
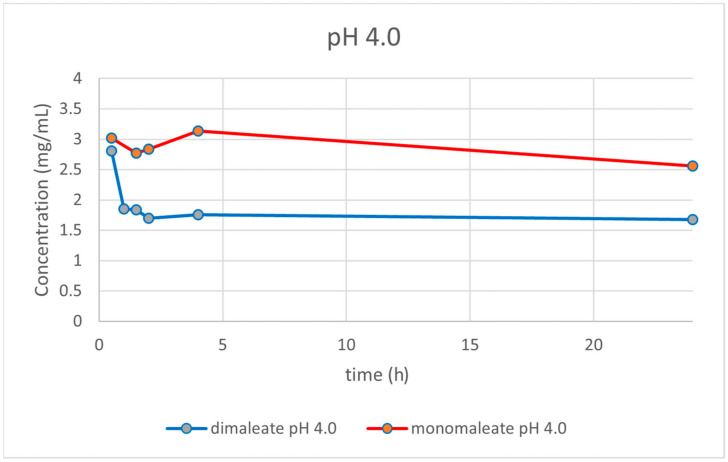
Dissolution testing of glasdegib monomaleate and glasdegib dimaleate at pH = 4.0.

At pH = 4.0 (Figure 15), the dissolution curves were different compared to those obtained at pH = 1.2. In this case, the solubility of both forms was dropping over time. After 24 h, glasdegib monomaleate had higher solubility (2.56 mg/mL) compared to the glasdegib dimaleate (1.68 mg/mL).

**Figure 16:**
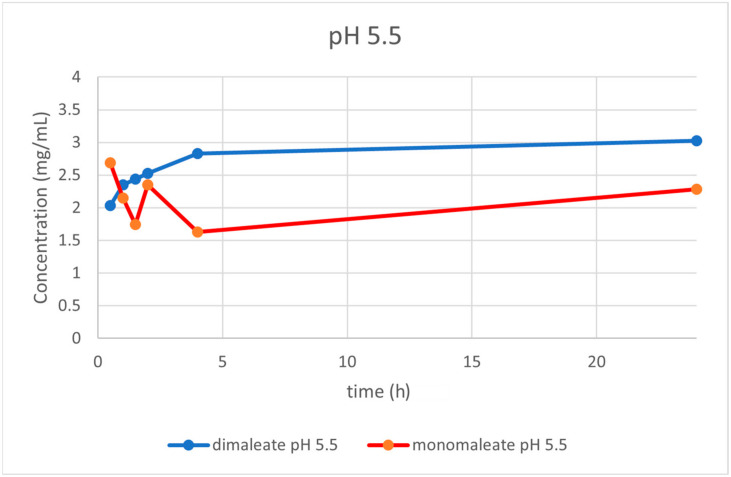
Dissolution testing of glasdegib monomaleate and glasdegib dimaleate at pH = 5.5.

At pH = 5.5 (Figure 16) the dissolution curves were similar compared to those obtained at pH = 1.2, slightly rising over time. After 24 h, glasdegib dimaleate had higher solubility (3.02 mg/mL) compared to the glasdegib monomaleate (2.28 mg/mL).

**Figure 17:**
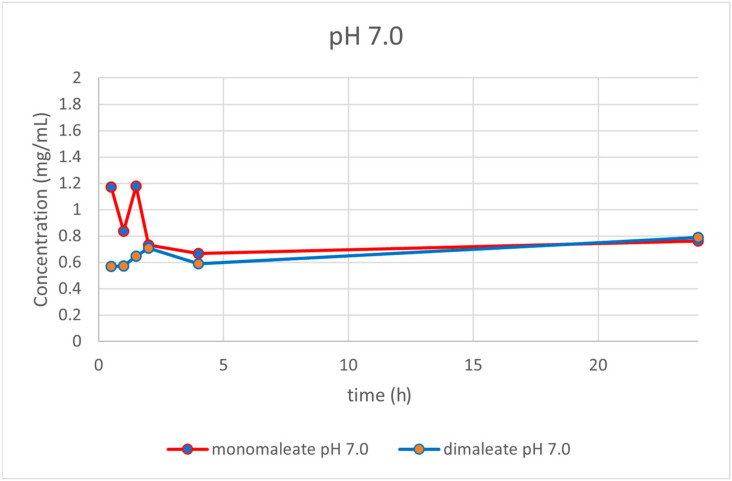
Dissolution testing of glasdegib monomaleate and glasdegib dimaleate at pH = 7.0.

At pH = 7.0 (Figure 17), the dissolution curves revealed that solubility was rising slightly over time after 4 h for both forms. After 24 h, glasdegib monomaleate had slightly lower solubility (0.76 mg/mL) compared to the glasdegib dimaleate (0.79 mg/mL).

## 4. Discussion

The formation of novel maleic acid containing a solid form of glasdegib was confirmed via PXRD, IR, Raman and DSC analysis. Indeed, notable differences in the PXRD diffractogram (Figure 2), IR and Raman spectra (Figure 3, Figure 4, Figure 5 and Figure 6), as well as in the DSC thermograms, were observed (Figure 7) between the known glasdegib monomaleate form and the novel form. As revealed by the solution ^1^H NMR analysis, the novel solid form had 2:1 stoichiometry between maleic acid and glasdegib. However, the determined stoichiometry of the formed solid brought some ambiguity related to the ionization state of the novel form.

In the past, the p*K*a difference between base and acid (Δp*K*a = p*K*a _protonated base_ − p*K*a _acid_) was established as one of the key parameters that defines the likelihood of salt formation. In general, it is considered that complexes with Δp*K*a > 4 form as salts, while Δp*K*a < −1 favors co-crystal formation. In the interim zone where −1 < Δp*K*a < 4, both types of species can be formed. Nevertheless, the likelihood of co-crystal formation is higher in the lower part of the scale close to or below Δp*K*a = 0 [39]. In the zone where Δp*K*a is between 0 and 3, a continuum between neutral and ionized species exits, which can provide acid–base complexes with mixed ionization states [40]. Very recently, such evidence was provided experimentally using ssNMR [41] and single-crystal X-ray determination [42] in the pharmaceutical field on tenofovir alafenamide fumarate.

Glasdegib contains two basic sites that can be protonated by an acid: methylpiperidine nitrogen and benzimidazole nitrogen (Figure 1). The reported p*K*a values of glasdegib (6.1 for methylpiperidine nitrogen and 1.7 for benzimidazole nitrogen) [24,25] and maleic acid (p*K*_a1_ = 1.9 and p*K*_a2_ = 6.3) [29] give Δp*K*a values of 4.2 in the case of the methylpiperidine nitrogen/maleic acid pair and −0.2 for the benzimidazole nitrogen/maleic acid pair (more acidic proton in maleic acid is considered; Table 4). The reported p*K*a values for glasdegib [24,25] do not provide information on which solvent they acquired, which brings some ambiguity to the Δp*K*a calculation, because p*K*a values change in different solvents. Therefore, we also calculated the p*K*a values for glasdegib and maleic acid using the ChemAxon Marvin Suite [43] in order to obtain solvent bias-free p*K*a and Δp*K*a values. The calculation using the ChemAxon Marvin Suite provided p*K*a values of 6.67 (methylpiperidine nitrogen) and 3.01 (benzimidazole nitrogen) for glasdegib, while p*K*a values of 2.85 and 5.75 were obtained for maleic acid. The calculated p*K*a values were 3.82 in the case of the methylpiperidine nitrogen/maleic acid pair and 0.16 for the benzimidazole nitrogen/maleic acid pair (Table 4). Thus, the calculated p*K*a data fit well with the measured data, and the obtained Δp*K*a values are similar for the reported and calculated values.

Based on the calculated Δp*K*a values for the glasdegib–maleic acid pair, it is obvious that in the case of glasdegib monomaleate, salt will be formed on methylpiperidine nitrogen because Δp*K*a = ca. 4 (Table 4). A similar situation can be anticipated in the case of glasdegib dimaleate, where one maleic acid will protonate the methylpiperidine nitrogen, whereas the second maleic acid could either act as a co-former to form a co-crystal, or form a salt with the benzimidazole nitrogen of glasdegib. The calculated probability for the formation of salt on the benzimidazole nitrogen **P(A^−^B^+^)** with the second maleic acid using Cruz-Cabeza equations [39] show that benzimidazole nitrogen should remain unprotonated (the probability of salt formation only 25–31%, Table 5), while the probability of co-crystal formation **P(AB)** is three times higher (Table 5). Therefore, the obtained glasdegib dimaleate should be a mixture of salt and co-crystal species: (maleic acid)^−^∙(maleic acid)∙(glasdegib)**^+^** = A^−^AB^+^, rather than a double-salt form: (maleic acid)^−^∙(maleic acid)**^−^**∙(glasdegib)**^+ +^** = A^−^A^−^B^++^ (Table 5).

To further confirm the structure of glasdegib monomaleate and glasdegib dimaleate, a single-crystal X-ray analysis of both forms was performed. As expected, it confirmed that glasdegib monomaleate exists in salt form with protonated methylpiperidine nitrogen. In the case of glasdegib dimaleate, it affirmed an interesting observation from the ^15^N ssNMR study that this complex exists as a double salt with protonated methylpiperidine nitrogen and benzimidazole nitrogen.

The confirmed double-salt structure (A^−^A^−^B^++^), instead of a salt-co-crystal species structure (A^−^AB^+^), for glasdegib dimaleate by ^15^N ssNMR and single-crystal X-ray analysis is surprising based on the p*K*a values of this acid–base pair. This outcome might be attributed to the specific choice of solvents used in the preparation of glasdegib dimaleate, since it is known that the proton transfer process can be solvent-dependent [44] and the choice of solvent plays a very important role in the synthesis of organic salts [45].

Until 2006, the maleate anion was used in 4.2% of marketed drugs containing basic active pharmaceutical ingredients [46]. Similarly, in the period of 2007–2016, the maleate anion was used in 4.3% of drugs that contained basic active pharmaceutical ingredients [47]. Some recent or well-known examples of marketed drugs that contain a maleate anion [48] are afatinib dimaleate (contains two basic sites with p*K*a values of 8.2 and 5.0) [49], neratinib monomaleate (contains two basic sites with p*K*a values of 7.65 and 4.66) [50], sunitinib monomaleate (contains one basic site with a p*K*a of 8.95) [51] and enalapril monomalate (contains one basic site with a p*K*a of 5.5) [52]. These data suggest that the maleate anion is used in active pharmaceutical ingredients that contain basic moieties with a p*K*a of 5 or higher, which facilitates salt-species formation. In this respect, glasdegib dimaleate represents and unusual example.

After the determination of the ionization state of glasdegib dimaleate by ^15^N ssNMR and single-crystal X-ray determination, the physiochemical properties of glasdegib dimaleate were investigated. The stress stability testing at 40 °C and 75% relative humidity demonstrated slightly better chemical and physical stability of glasdegib dimaleate compared to the monomaleate form. Moreover, dissolution testing and solubility determination at pH values of 1.2 (Figure 14) and 7.0 (Figure 17) showed good comparability of both forms, while lower solubility of dimaleate was observed at a pH of 4.0 (Figure 15) and higher solubility was observed at a pH of 5.5 (Figure 16). These properties of glasdegib dimaleate ascertain its suitability for the development of pharmaceutical dosage forms.

## 5. Conclusions

In summary, for the first time, glasdegib dimaleate was prepared and fully characterized using spectroscopic and thermal analyses, which demonstrated that glasdegib dimaleate exists in double-salt form. This is a surprising finding based on the known p*K*_a_ values of glasdegib and maleic acid in the literature. This comparative study of the physicochemical properties of both forms suggested that glasdegib dimaleate has similar aqueous solubility and slightly better stability under stress conditions. These properties affirm the glasdegib dimaleate form as a suitable candidate for the development of pharmaceutical preparation. Finally, the presented study demonstrates how unpredictable the formation of a specific pharmaceutical solid can be in terms of its chemical structure.

## 6. Patents

This work is based on our International Patent Application WO 2021191278 A1, 30 September 2021.

## Figures and Tables

**Table 1:** Crystallographic data for glasdegib monomaleate and glasdegib dimaleate.

	Glasdegib Monomaleate	Glasdegib Dimaleate
CCDC number	2,180,664	2,180,665
Formula	C_25_H_26_N_6_O_5_	C_29_H_30_N_6_O_9_
*M*_r_	490.52	606.59
*T* (K)	150.00(10)	150.00(10)
Crystal system	monoclinic	orthorhombic
Space group	*P*2_1_	*P*2_1_2_1_2_1_
*a* (Å)	9.7312(3)	10.3645(2)
*b* (Å)	12.3780(3)	14.5078(3)
*c* (Å)	10.5764(3)	19.2377(3)
*α* (°)	90	90
*β* (°)	113.776(3)	90
*γ* (°)	90	90
Volume (Å^3^)	1165.83(6)	2892.71(10)
Z	2	4
*D*_c_ (g/cm^3^)	1.397	1.393
*μ* (mm^−1^)	0.827	0.885
*F* (000)	516.0	1272.0
Reflections collected	8533	10926
Independent reflections (*R*_int_)	4415 (0.0196)	5775 (0.0376)
Data/restraints/parameters	4415/3/342	5775/4/419
*R*, *wR*_2_ [*I* > 2σ(*I*)] ^a^	0.0284, 0.0718	0.0436, 0.1047
*R*, *wR*_2_ (all data) ^a^	0.0304, 0.0737	0.0499, 0.1104
GOF, *S* ^b^	1.039	1.052
Largest diff. peak/hole/e Å^−3^	0.15/−0.16	0.27/−0.21
Flack parameter	−0.10(8)	0.23(13)

^a^*R* = ∑||*F*_o_| − |*F*_c_||/∑|*F*_o_|, *wR*_2_ = {∑[*w*(*F*_o_^2^ − *F*_c_^2^)^2^]/∑[*w*(*F*_o_^2^)^2^]}^1/2^. ^b^ *S* = {∑[(*F*_o_^2^ − *F*_c_^2^)^2^]/(*n*/*p*}^1/2^, where *n* is the number of reflections and *p* is the total number of refined parameters.

**Table 2:** Hydrogen bonds for glasdegib monomaleate and glasdegib dimaleate [Å and °].

D–H⋯A	*d*(D–H)	*d*(H⋯A)	*d*(D⋯A)	<(DHA)
glasdegib monomaleate				
N1–H1⋯O3^i^	0.885(13)	2.033(15)	2.892(2)	164(2)
N4–H4⋯O2	0.98(2)	1.78(3)	2.687(2)	151(2)
N6–H6⋯O5^i^	0.90(3)	1.87(3)	2.762(2)	171(3)
O3–H3A⋯O4	1.16(3)	1.25(3)	2.403(2)	177(3)
C3–H3⋯O2^i^	0.95	2.54	3.458(3)	162.7
C7–H7⋯O1	0.95	2.25	2.863(3)	121.7
C11–H11B⋯N3^ii^	0.99	2.42	3.407(3)	172.5
C12–H12⋯O4^i^	1.00	2.25	3.211(2)	161.9
C18–H18⋯O1^iii^	0.95	2.56	3.302(3)	135.2
C23–H23⋯N5	0.95	2.54	3.274(3)	134.3
glasdegib dimaleate				
N1–H1⋯O4^i^	0.891(13)	1.929(14)	2.820(3)	177(4)
N2–H2⋯O5^i^	0.886(13)	2.122(15)	2.999(3)	170(3)
N4–H4⋯O2	0.99(4)	1.76(4)	2.703(3)	158(3)
N5–H5⋯O6	0.871(13)	1.872(15)	2.736(4)	171(4)
N5–H5⋯O7	0.871(13)	2.56(3)	3.192(4)	130(3)
N6–H6⋯O8^i^	0.877(13)	2.36(2)	3.153(4)	150(3)
N6–H6⋯O9^i^	0.877(13)	2.05(3)	2.772(4)	140(3)
O4–H3A⋯O3	1.17(5)	1.23(5)	2.397(3)	170(4)
O8–H7A⋯O7	1.15(5)	1.24(6)	2.386(4)	177(5)
C7–H7⋯O1	0.95	2.22	2.842(4)	122.4
C9–H9⋯O5^ii^	1.00	2.50	3.326(4)	139.6
C12–H12⋯O5^i^	1.00	2.45	3.228(4)	134.6
C13–H13B⋯O6	0.99	2.42	3.364(4)	159.0
C21–H21⋯O7	0.95	2.50	3.244(4)	134.8
C28–H28⋯N3^iii^	0.95	2.60	3.404(5)	142.7

Symmetry codes for glasdegib monomaleate: (i) *x*, *y*, 1 + *z*; (ii) 1 − *x*, ½ + *y*, 2 − *z*; (iii) *x*, 1 + *y*, *z*, and for glasdegib dimaleate: (i) ½ − *x*, 1 − *y*, ½ + *z*; (ii) 1 − *x*, −½ + *y*, ½ − *z*; (iii) *x*, *y*, −1 + *z*.

**Table 3:** Stability of glasdegib monomaleate and glasdegib dimaleate.

Form Type and Testing Time Point	Chiral Purity ^1^ [Area%]	Purity ^2^ [Area%]
glasdegib monomaleate, t = 0	100.00	99.86
glasdegib monomaleate, t = 1 month	99.96	99.81
glasdegib monomaleate, t = 2 months	99.96	99.73
glasdegib monomaleate, t = 3 months	99.96	99.72
glasdegib dimaleate, t = 0	100.00	99.98
glasdegib dimaleate, t = 1 month	100.00	99.97
glasdegib dimaleate, t = 2 months	100.00	99.93
glasdegib dimaleate, t = 3 months	100.00	99.90

^1^ Determined via chiral HPLC method. ^2^ Determined via UHPLC method.

**Table 4:** Calculated Δp*K*a values for glasdegib–maleic acid complex considering glasdegib methylpiperidine nitrogen/maleic acid pair and benzimidazole nitrogen/maleic acid pair.

p*K*_a_ Protonated Base	p*K*_a1_ Acid [29]	Δp*K*a = p*K*a (Protonated Base) − p*K*_a1_ (Acid) ** [39]
6.1 (methylpiperidine nitrogen) [24,25]	1.9 [29]	4.2
1.7 (benzimidazole nitrogen) [24,25]	1.9 [29]	−0.2
6.67 (methylpiperidine nitrogen) * [43]	2.85 * [43]	3.82
3.01 (benzimidazole nitrogen) * [43]	2.85 * [43]	0.16

* Calculated using ChemAxon Marvin Suite 17.28.0. ** Calculation performed for more acidic proton in maleic acid.

**Table 5:** Calculated probability for salt or co-crystal formation for the protonation of benzimidazole nitrogen with the second maleic acid molecule in the glasdegib–maleic acid 1:2 complex.

	Δp*K*a	P(A^−^B^+^) ^1^ (%)	P(AB) ^1^ (%)
Reported p*K*a values.	−0.2	24.6	75.4
Calculated p*K*a values	0.16	30.7	69.3

^1^ Calculated probability (P) value based on Cruz-Cabeza equations [39]: P (AB, %) = −17∙Δp*K*a + 72 and P (A^−^B^+^, %) = 17∙Δp*K*a + 28.

## Data Availability

Not applicable.

## Supplementary Materials

The following supporting information can be downloaded at: https://www.mdpi.com/article/10.3390/pharmaceutics14081641/s1, Figure S1: DSC and TGA thermogram of glasdegib monomaleate; Figure S2: DSC and TGA thermogram of glasdegib dimaleate; Figure S3: DSC and TGA thermogram of glasdegib base; Figure S4: DSC and TGA thermogram of glasdegib dihydrochloride hydrate; Figure S5: IR spectrum of glasdegib monomaleate, 1800–400 cm^−1^; Figure S6: IR spectrum of glasdegib dimaleate, 1800–400 cm^−1^; Figure S7: IR spectrum of glasdegib base, 4000–400 cm^−1^; Figure S8: IR spectrum of glasdegib base, 1800–400 cm^−1^; Figure S9: IR spectrum of glasdegib dihydrochloride hydrate, 4000–400 cm^−1^; Figure S10: IR spectrum of glasdegib dihydrochloride hydrate, 1800–400 cm^−1^; Figure S11: IR spectrum of maleic acid, 4000–400 cm^−1^; Figure S12: IR spectrum of maleic acid, 1800–400 cm^−1^; Figure S13: Raman spectrum of glasdegib base; Figure S14: Raman spectrum of glasdegib dihydrochloride; Figure S15: Raman spectrum of maleic acid; Figure S16: Measured PXRD pattern of glasdegib monomaleate powder; Figure S17: Calculated PXRD pattern from the single-crystal structure of glasdegib monomaleate; Figure S18: Measured PXRD pattern of glasdegib dimaleate powder; Figure S19: Calculated PXRD pattern from the single-crystal structure of glasdegib dimaleate; Figure S20: Measured PXRD pattern of glasdegib base powder; Figure S21: Measured PXRD pattern of glasdegib dihydrochloride powder; Figure S22: Measured PXRD pattern of glasdegib dimaleate powder exposed to stress conditions for 2 months; Figure S23: Measured PXRD pattern of glasdegib monomaleate powder exposed to stress conditions for 1 month; Figure S24: Ultra-high-performance liquid chromatogram of glasdegib for determination of solubility; Figure S25: Chiral high-performance liquid chromatogram for determination of chiral purity; Figure S26: ^1^H echo MAS NMR spectra of glasdegib base, glasdegib dihydrochloride hydrate, glasdegib monomaleate and glasdegib dimaleate; Figure S27: ^1^H-NMR spectrum of glasdegib monomaleate in MeOD at 500 MHz; Figure S28: ^13^C-NMR spectrum of glasdegib monomaleate in MeOD at 125 MHz; Figure S29: ^1^H-NMR spectrum of glasdegib dimaleate in MeOD at 500 MHz; Figure S30: ^13^C-NMR spectrum of glasdegib dimaleate in MeOD at 125 MHz; Figure S31: MarvinSketch 17.28 calculated p*K*_a_ values for glasdegib; Figure S32: MarvinSketch 17.28 calculated p*K*_a_ values for maleic acid. CCDC 2180664 and 2180665 contain the supplementary crystallographic data for this paper. These data can be obtained free-of-charge via www.ccdc.cam.ac.uk/data_request/cif, by emailing data_request@ccdc.cam.ac.uk or by contacting The Cambridge Crystallography Data Centre, 12 Union Road, Cambridge CB2 1EZ, UK; fax: +44-1223-336033.
