# Supplementary material for: Glasdegib Dimaleate: Synthesis, Characterization and Comparison of Its Properties with Monomaleate Analogue
Source: Pharmaceutics. 2022 Aug 6;14(8):1641. doi: 10.3390/pharmaceutics14081641 (PMC9415664; doi:10.3390/pharmaceutics14081641)
Supplement: Supplementary file 1 [file pharmaceutics-14-01641-s001.zip › pharmaceutics-1830556-supplementary.pdf]

pharmaceutics

MDPI

Supplementary Materials: Glasdegib Dimaleate Form: Synthesis, Characterization and Comparison of its Properties with Monomaleate Analogue

Boris Peklar [1,2], Franc Perdih [3], Damjan Makuc [4,5], Janez Plavec [4,5], Jérôme Cluzeau [1], Zoran Kitanovski [1] and Zdenko Časar [1,6,*]

[1] Lek Pharmaceuticals d.d.; Sandoz Development Center Slovenia, Kolodvorska 27, 1234 Mengeš, Slovenia
[2] University of Maribor, Faculty of Chemistry and Chemical Engineering, Smetanova ulica 17, 2000 Maribor, Slovenia
[3] University of Ljubljana, Faculty of Chemistry and Chemical Technology, Večna pot 113, 1000 Ljubljana, Slovenia
[4] National Institute of Chemistry, Slovenian NMR Centre, Hajdrihova 19, SI-1001 Ljubljana, Slovenia
[5] EN-FIST Centre of Excellence, Trg Osvobodilne fronte 13, SI-1000 Ljubljana, Slovenia
[6] Faculty of Pharmacy, University of Ljubljana, Aškerčeva cesta 7, SI-1000 Ljubljana, Slovenia
* **Correspondence:** zdenko.casar@sandoz.com, zdenko.casar@ffa.uni-lj.si

Table of Contents

1. **DSC & TGA thermograms** **S2**
2. **Infrared spectra** **S4**
3. **Raman spectra** **S8**
4. **PXRD diffractograms** **S10**
5. **LC Chromatograms** **S14**
6. **ssNMR spectra** **S15**
7. **Solution ^{1}H- and ^{13}C-NMR spectra of glasdegib derivatives** **S16**
   7.1. Glasdegib monomaleate S16
   7.2. Glasdegib dimaleate S18
8. **Calculated pK_a data of glasdegib and maleic acid** **S20**

1. DSC & TGA thermograms

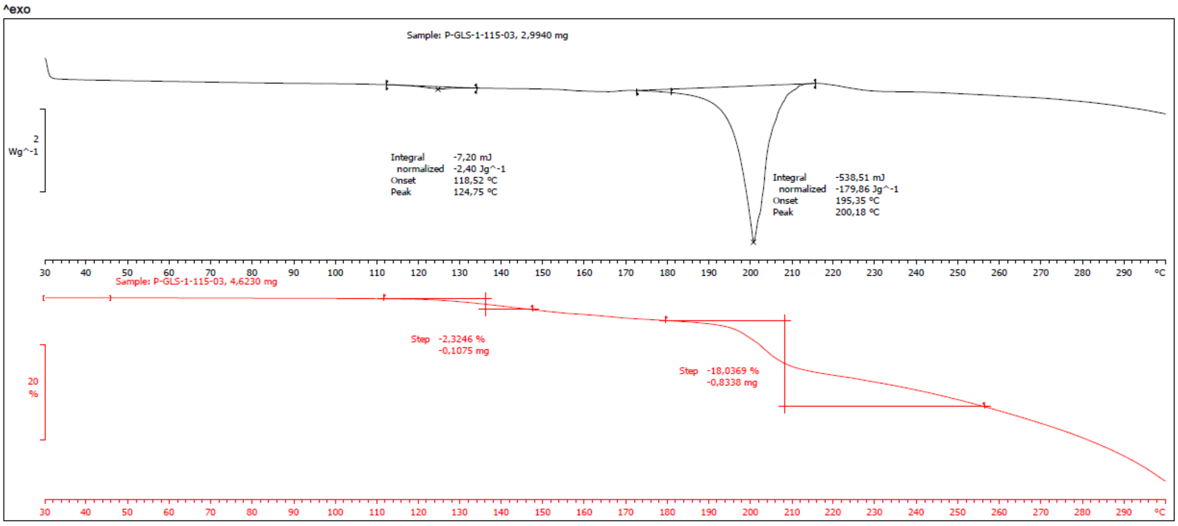

Figure S1: DSC & TGA thermogram of glasdegib monomaleate.

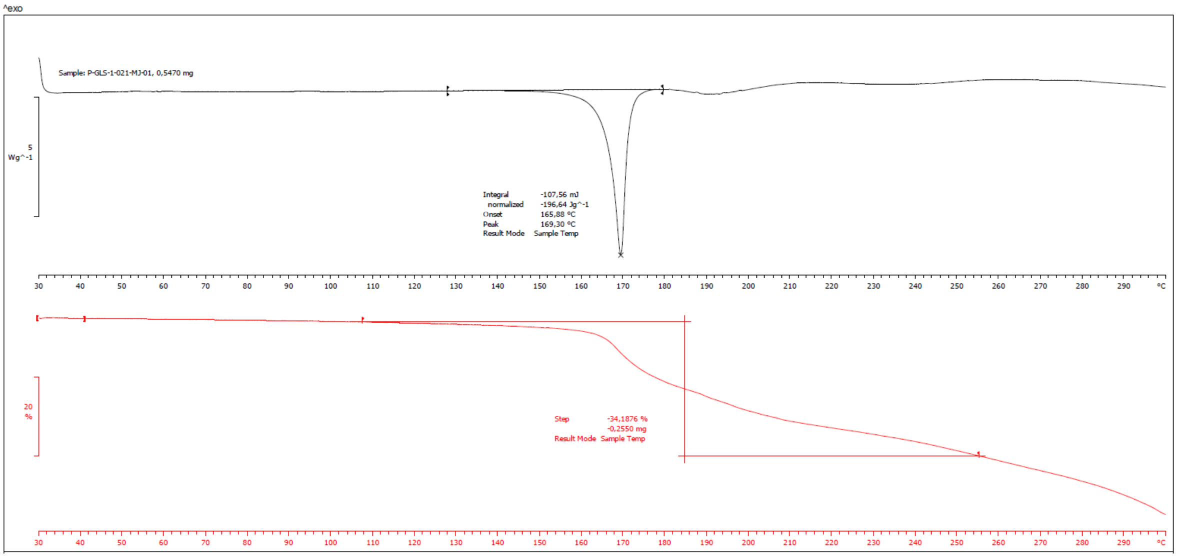

Figure S2: DSC & TGA thermogram of glasdegib dimaleate.

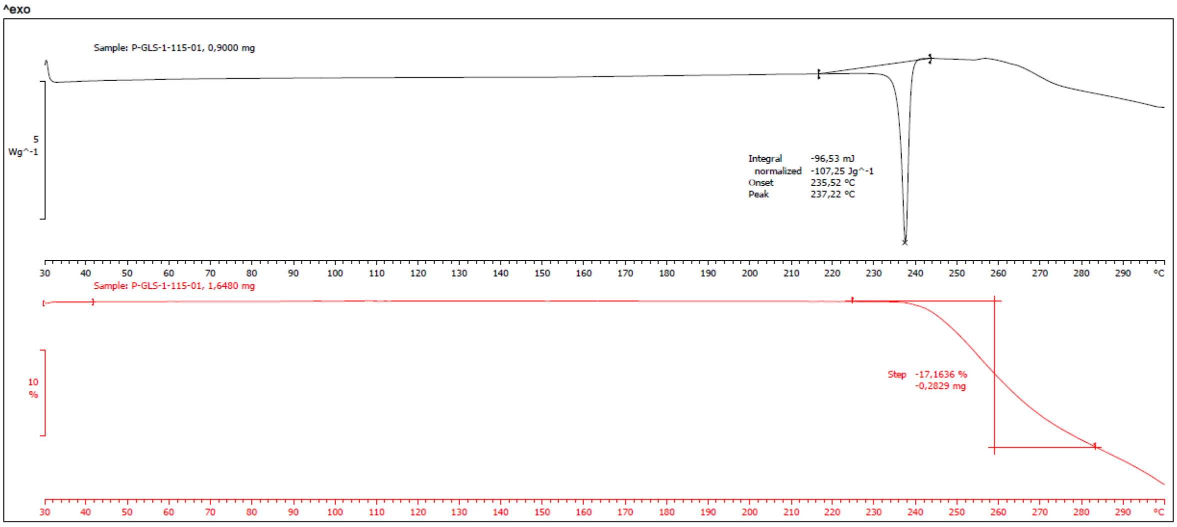

Figure S3: DSC & TGA thermogram of glasdegib base.

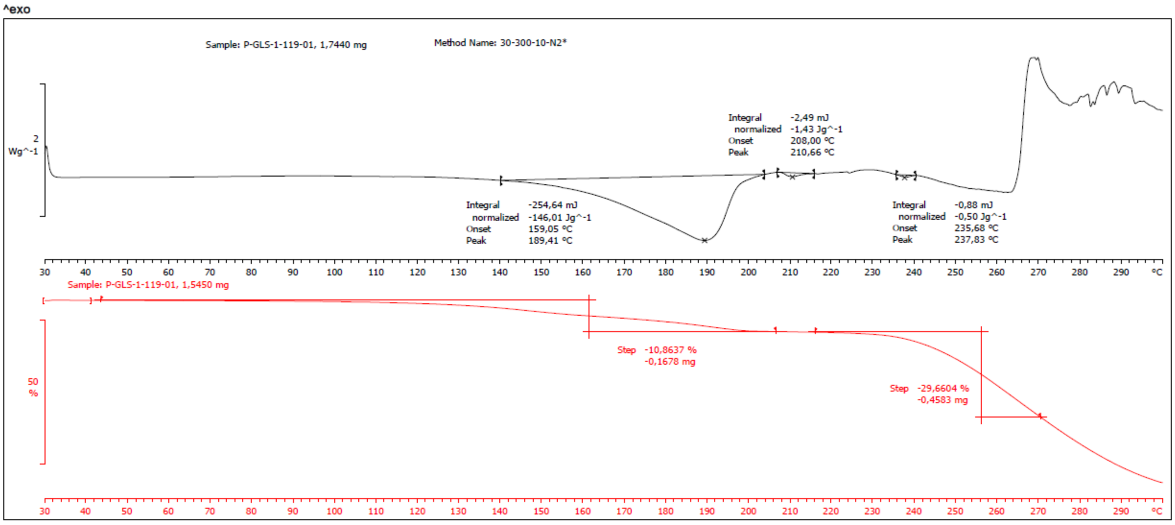

Figure S4: DSC & TGA thermogram of glasdegib dihydrochloride hydrate.

2. Infrared spectra

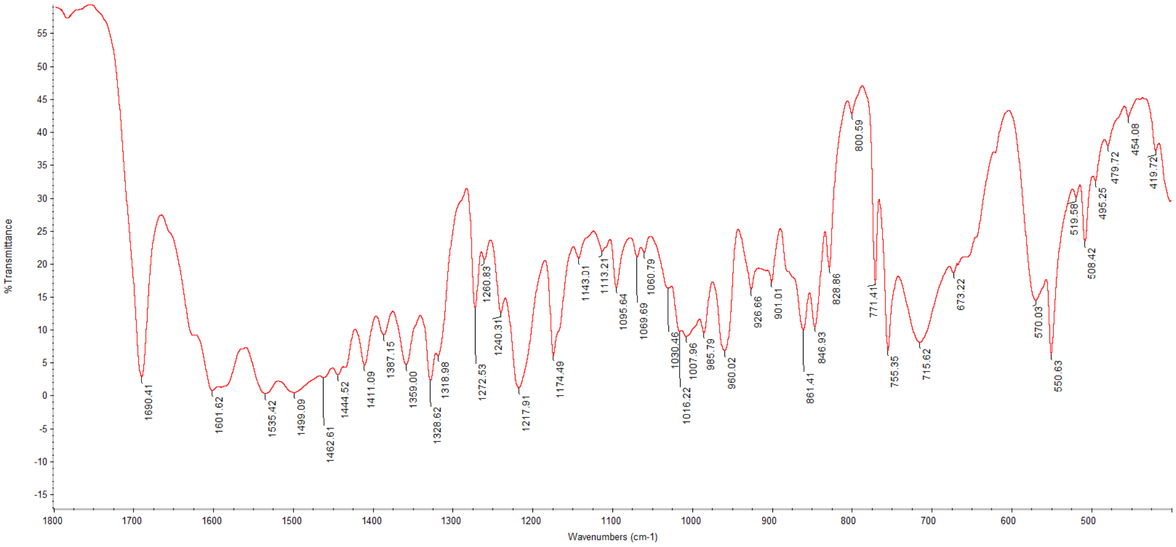

Figure S5: IR spectrum of glasdegib monomaleate 1800-400 cm-1.

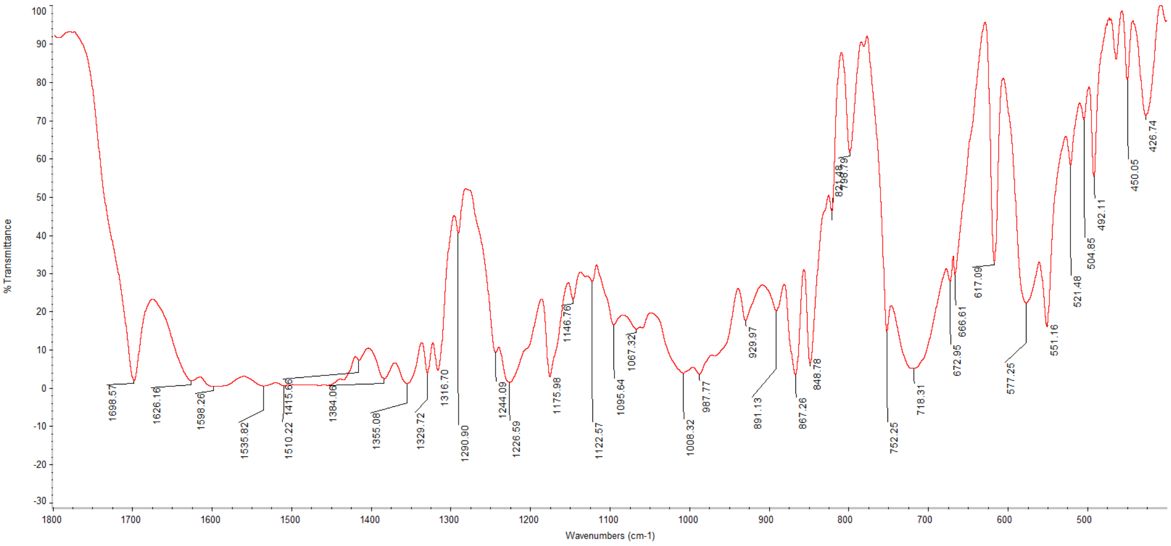

Figure S6: IR spectrum of glasdegib dimaleate 1800-400 cm-1.

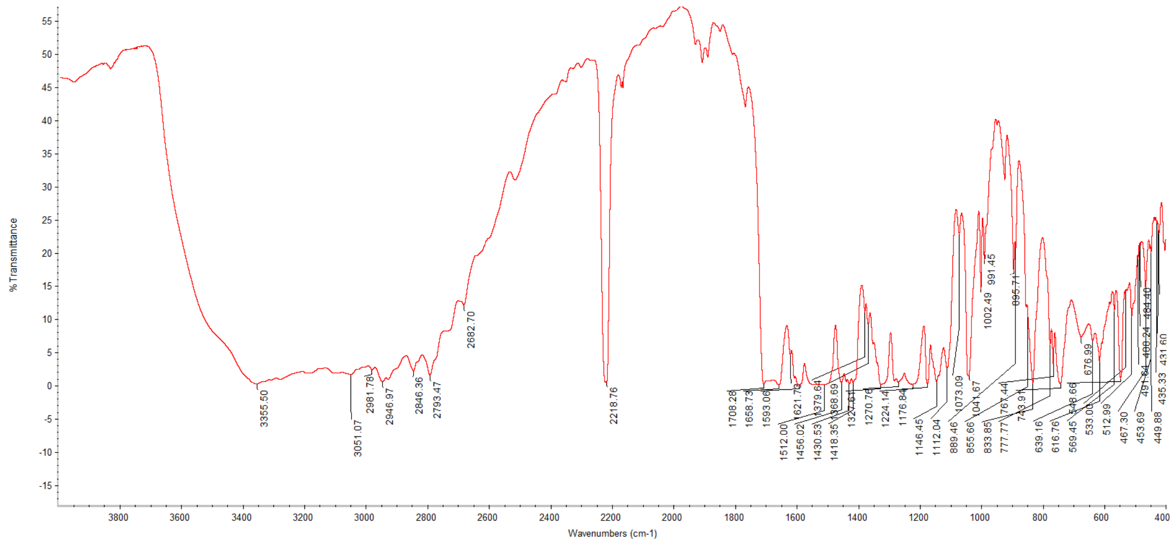

Figure S7: IR spectrum of glasdegib base 4000-400 cm-1.

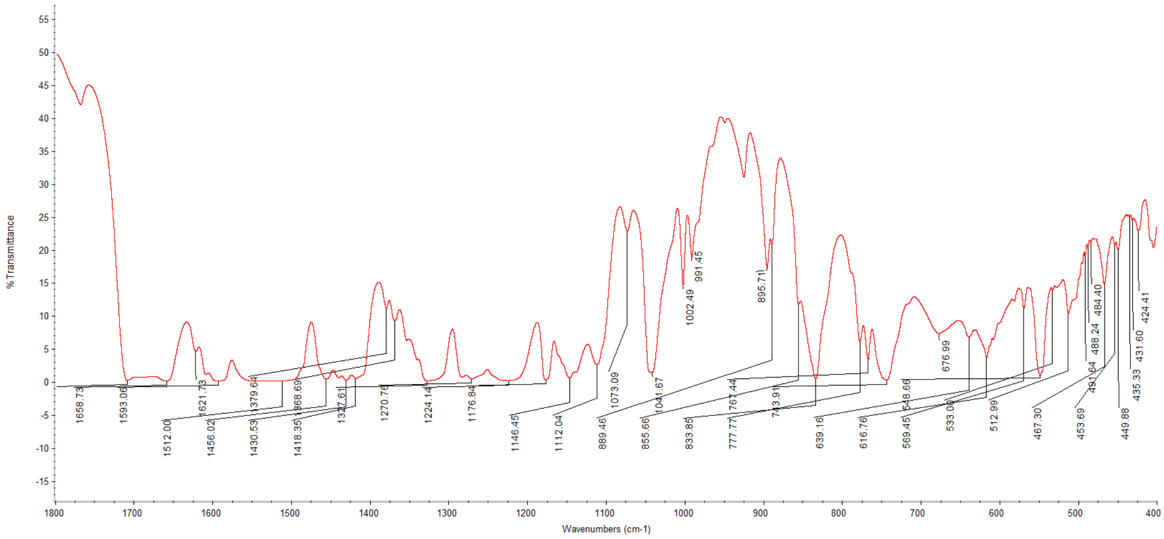

Figure S8: IR spectrum of glasdegib base 1800-400 cm-1.

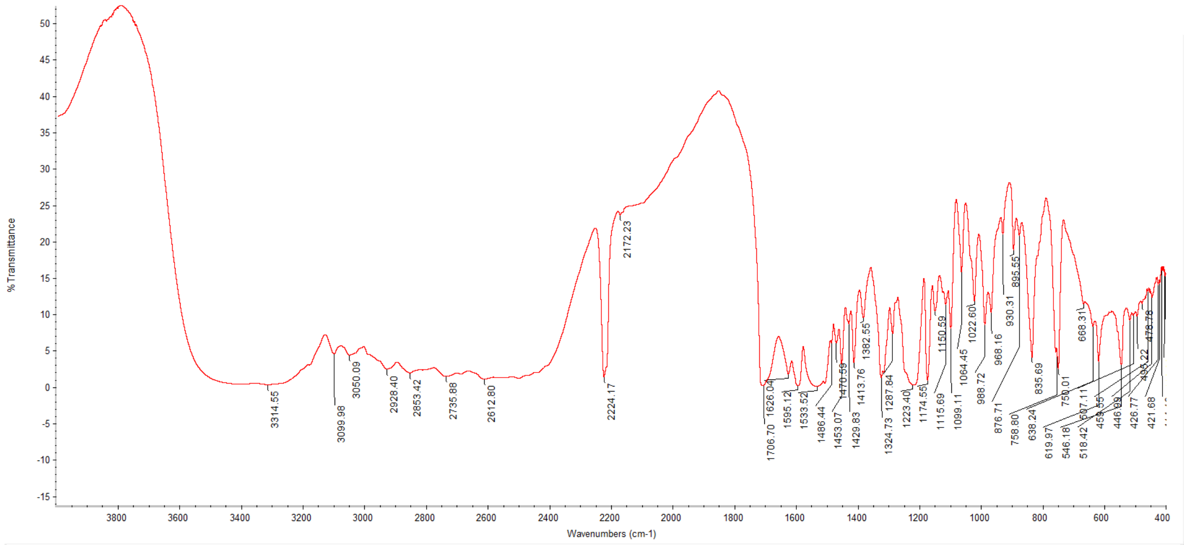

Figure S9. IR spectrum of glasdegib dihydrochloride hydrate 4000-400 cm^{-1}.

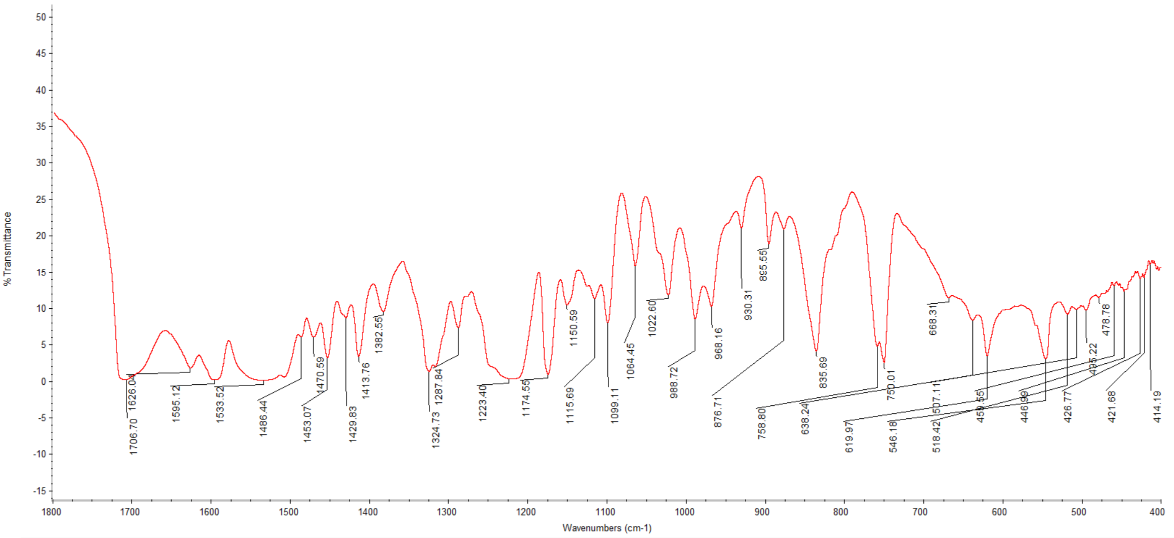

Figure S10. IR spectrum of glasdegib dihydrochloride hydrate 1800-400 cm^{-1}.

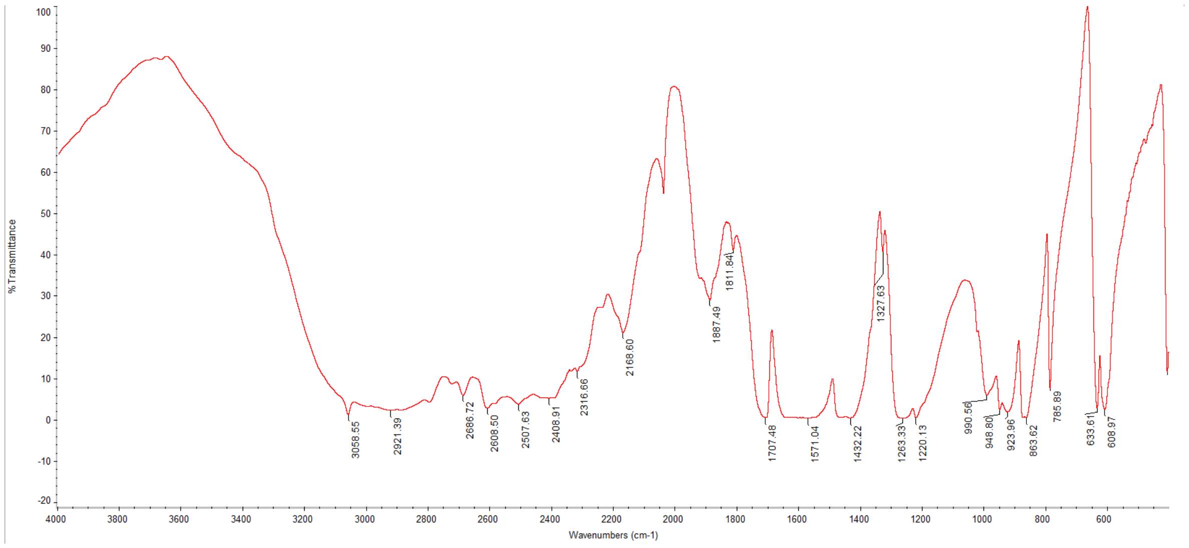

Figure S11. IR spectrum of maleic acid 4000-400 cm^{-1}.

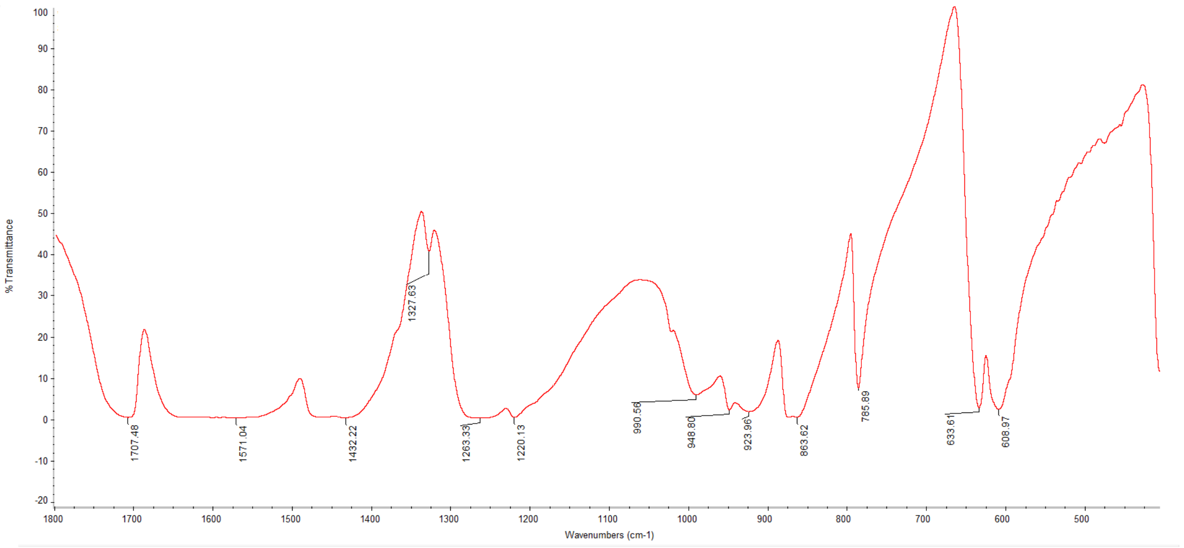

Figure S12. IR spectrum of maleic acid 1800-400 cm^{-1}.

3. Raman spectra

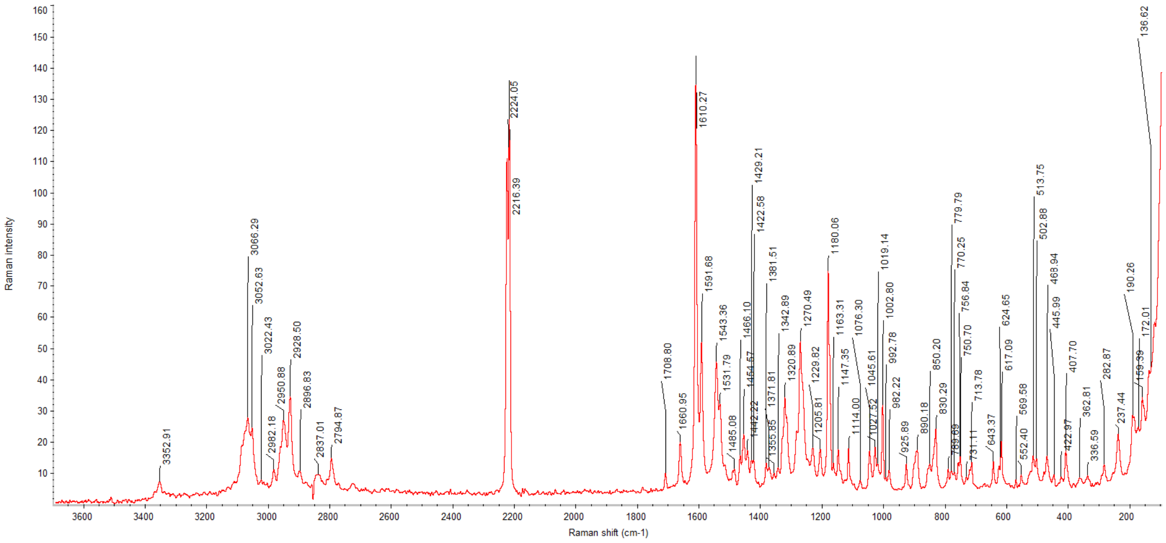

Figure S13. Raman spectrum of glasdegib base.

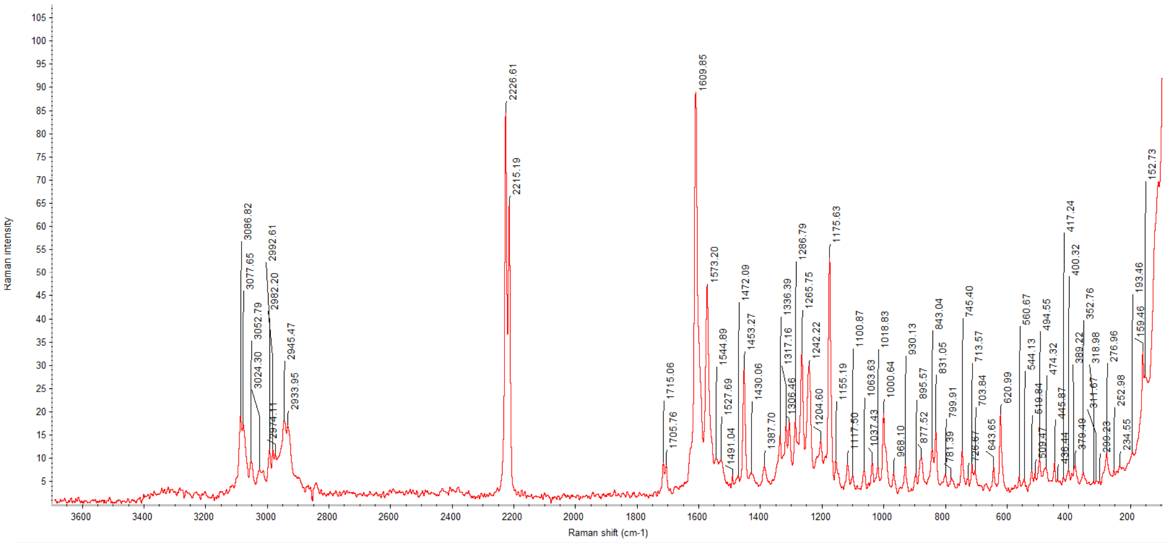

Figure S14. Raman spectrum of glasdegib dihydrochloride.

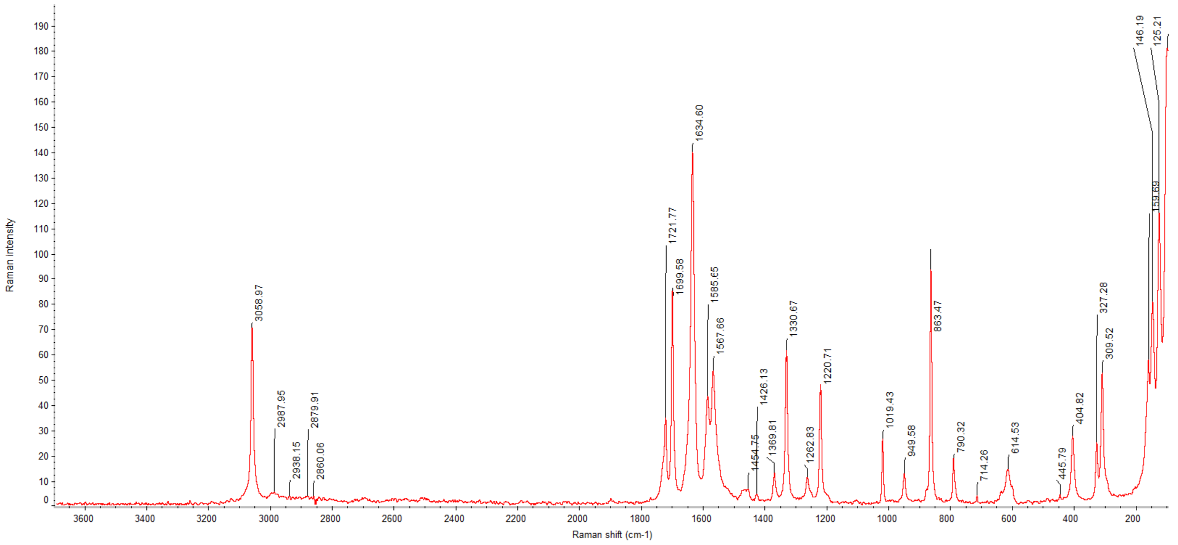

Figure S15. Raman spectrum of maleic acid.

4. PXRD diffractograms

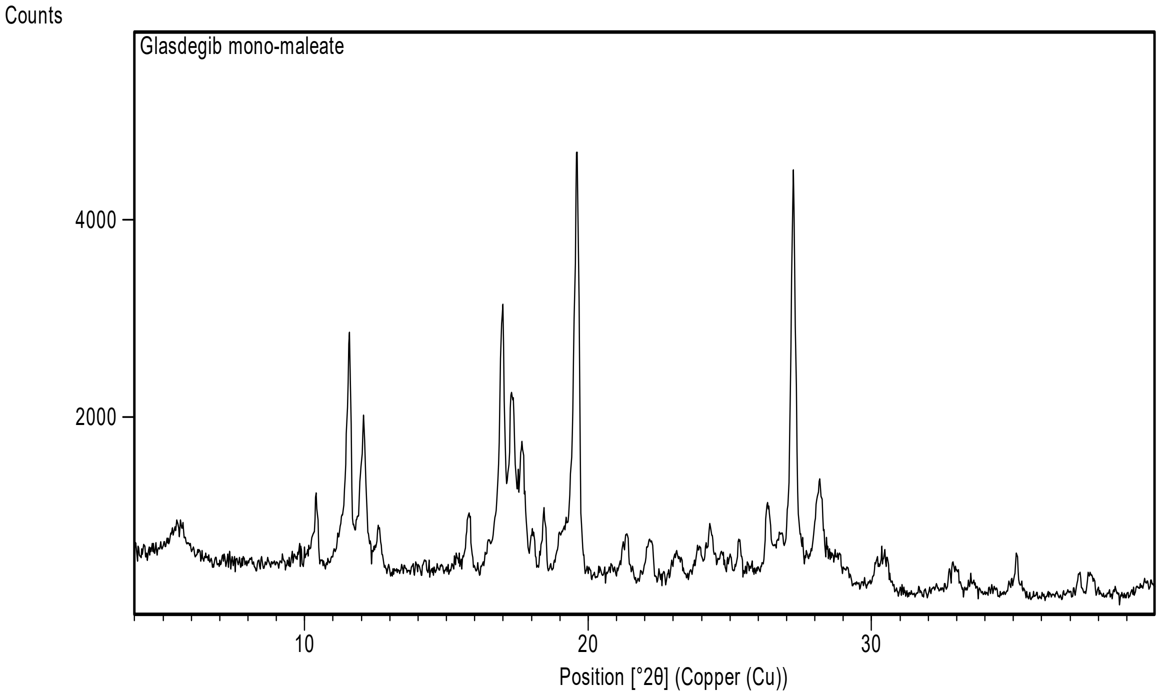

Figure S16. Measured PXRD pattern of glasdegib monomaleate powder.

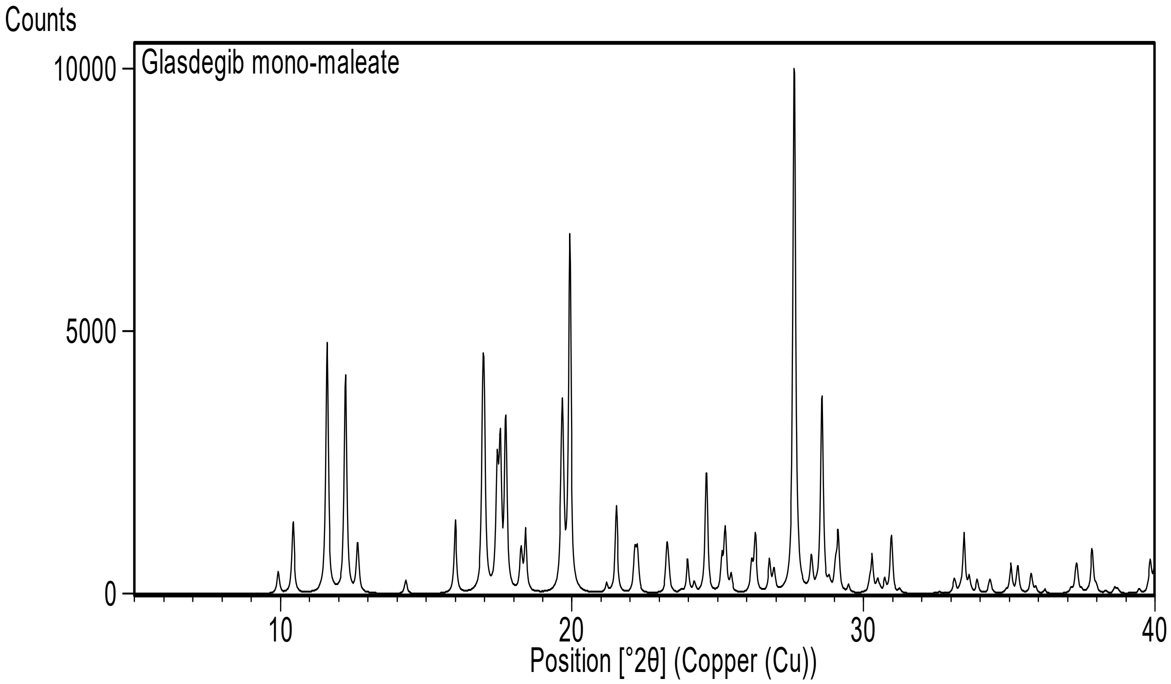

Figure S17. Calculated PXRD pattern from the single-crystal structure of glasdegib monomaleate.

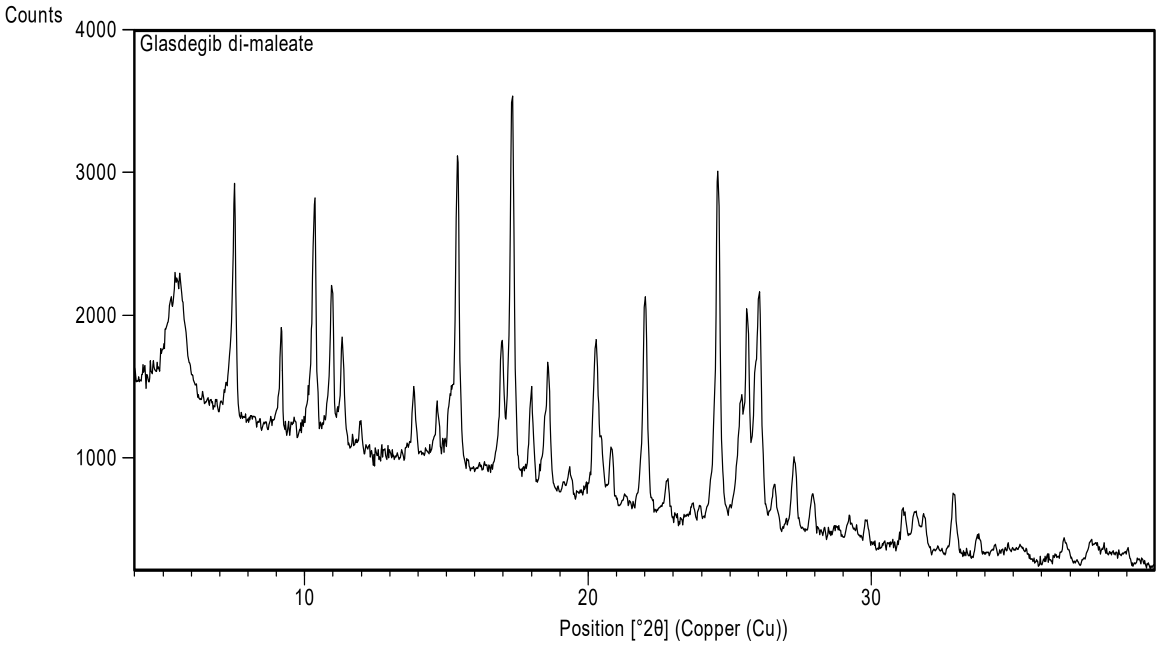

Figure S18. Measured PXRD pattern of glasdegib dimaleate powder.

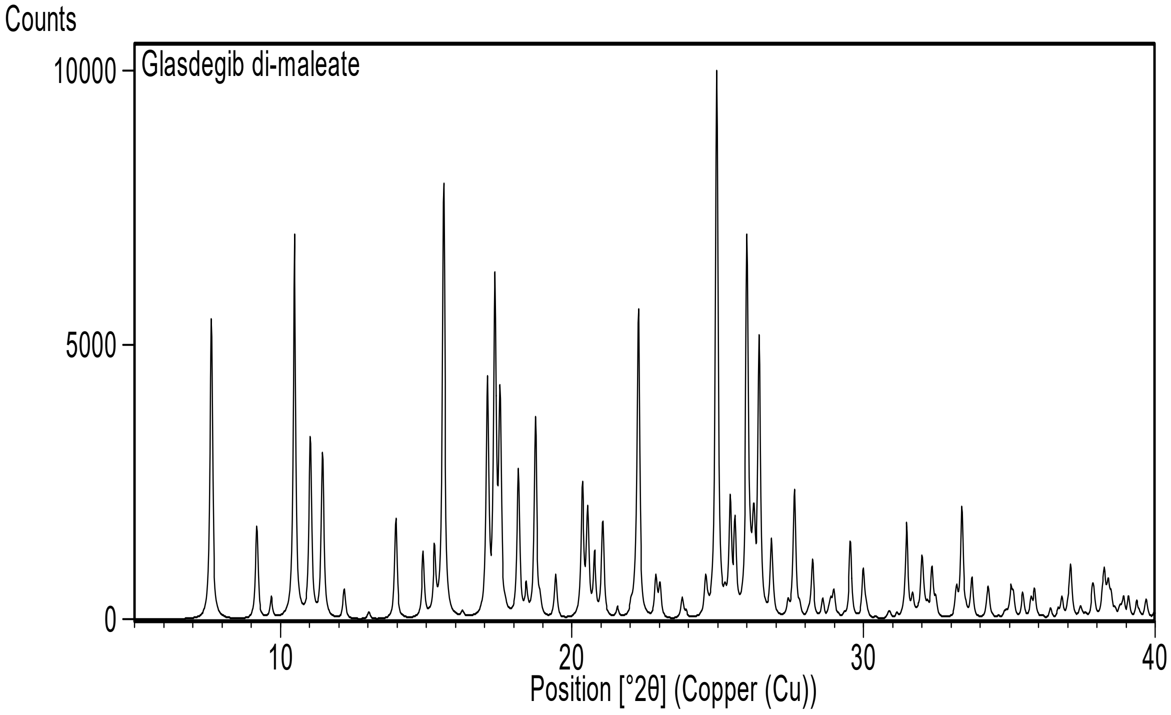

Figure S19. Calculated PXRD pattern from the single-crystal structure of glasdegib dimaleate.

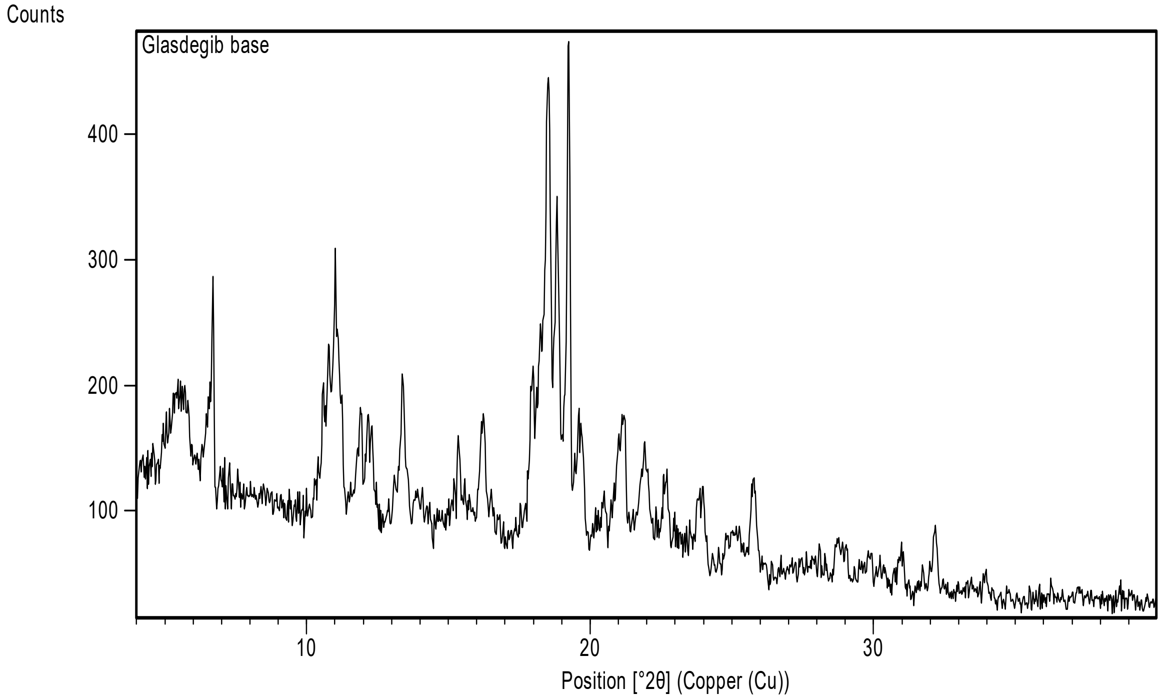

Figure S20. Measured PXRD pattern of glasdegib base powder.

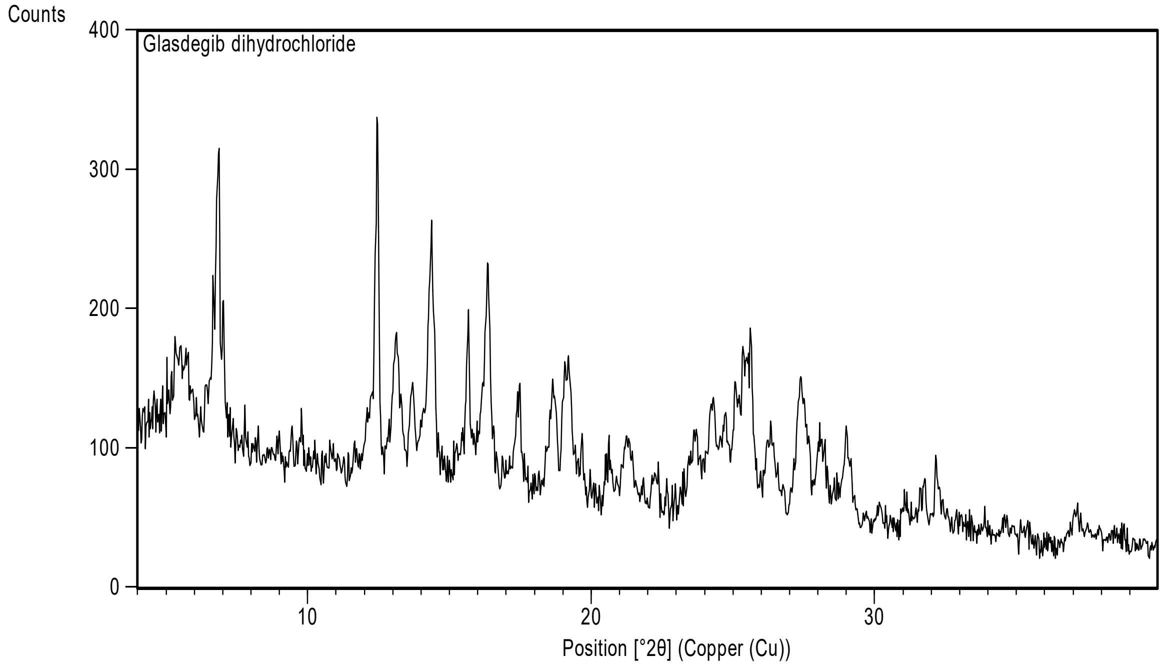

Figure S21. Measured PXRD pattern of glasdegib dihydrochloride powder.

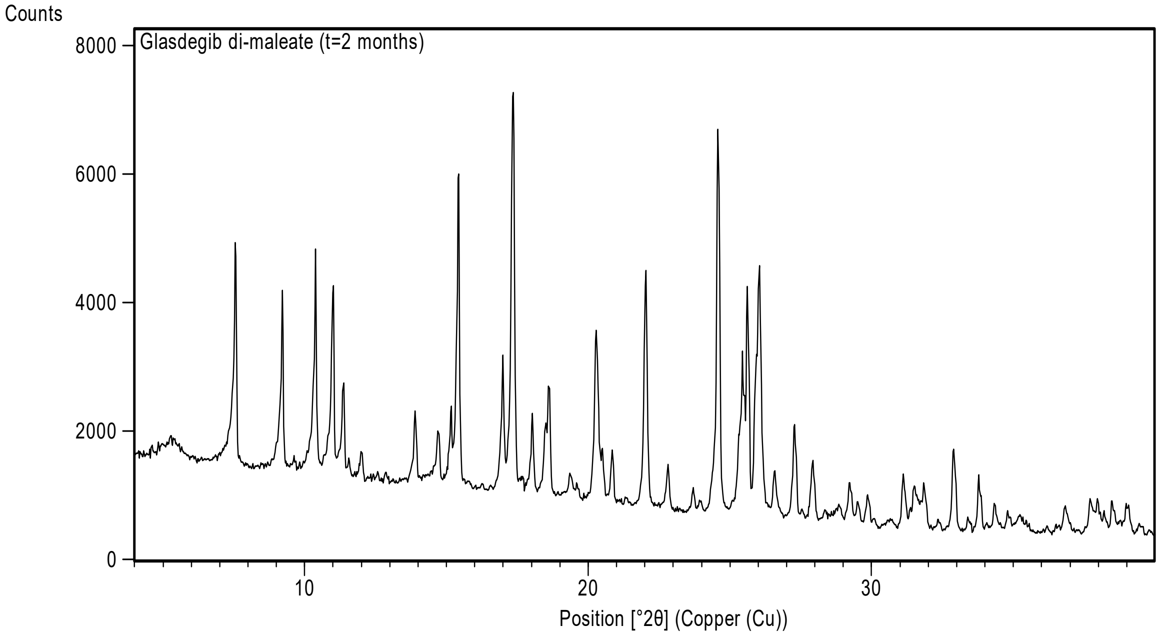

Figure S22. Measured PXRD pattern of glasdegib dimaleate powder exposed to stress conditions for 2 months.

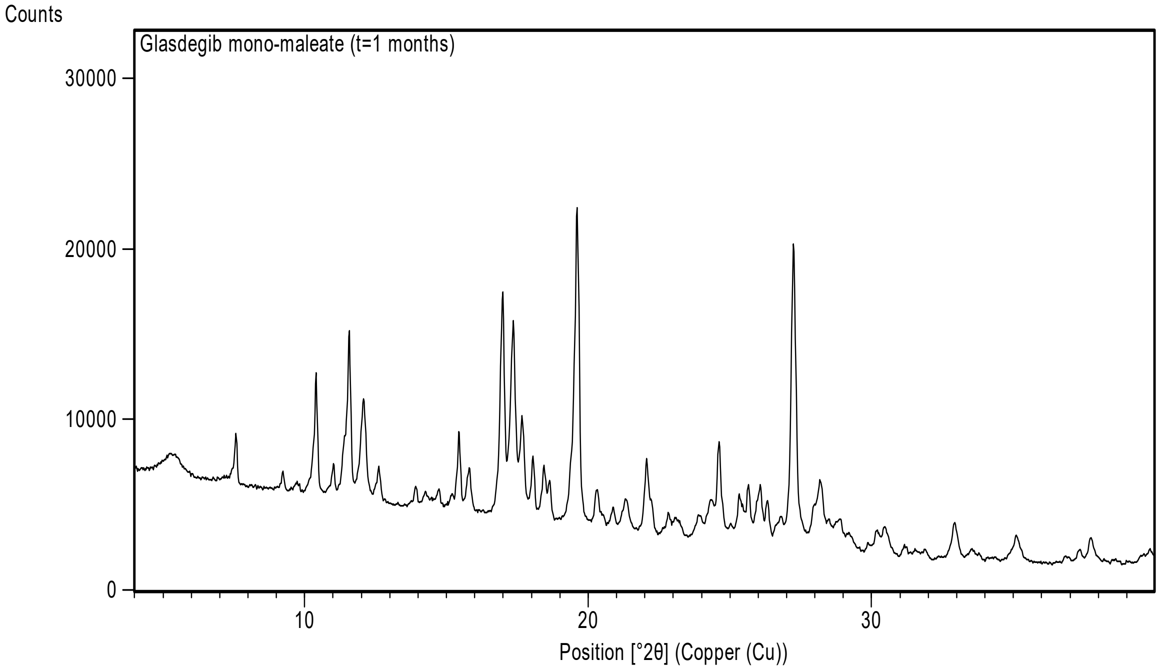

Figure S23. Measured PXRD pattern of glasdegib monomaleate powder exposed to stress conditions for 1 months.

5. LC Chromatograms

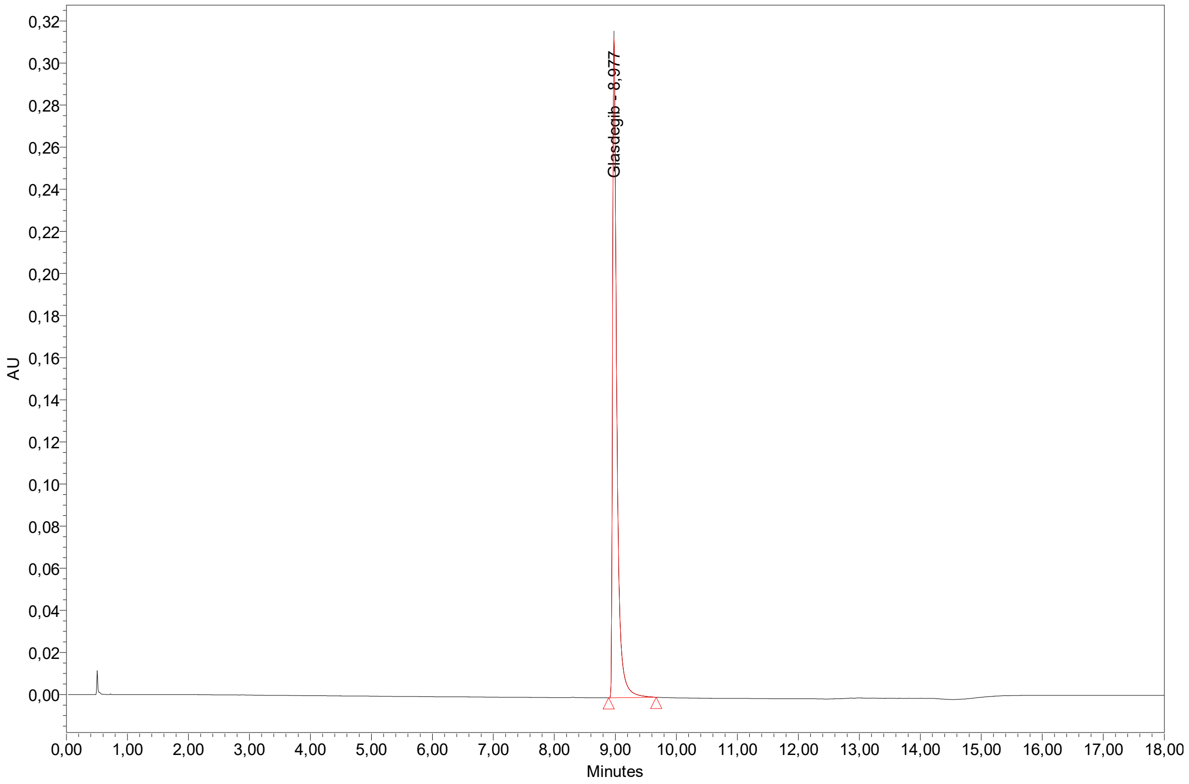

Figure S24. Ultra high performance liquid chromatogram of glasdegib for determination of solubility.

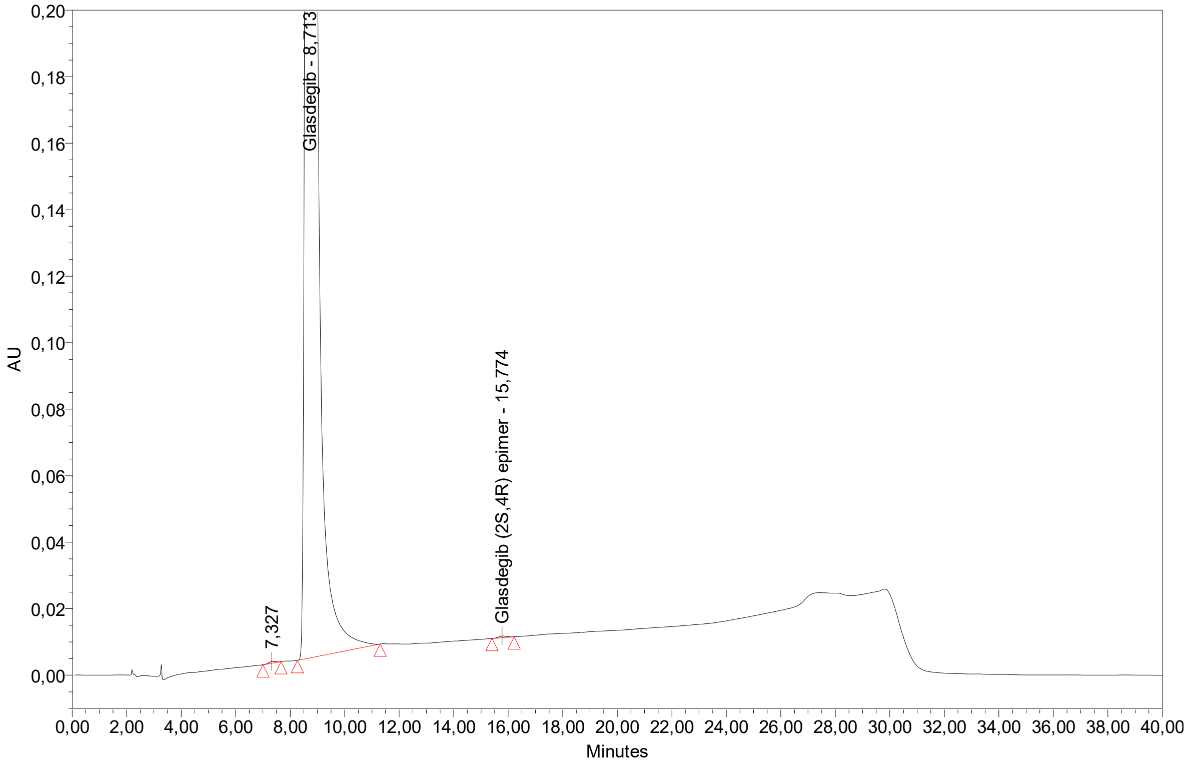

Figure S25. Chiral high performance liquid chromatogram for determination of chiral purity.

6. ssNMR spectra

^{1}H echo MAS NMR spectra of glasdegib derivatives

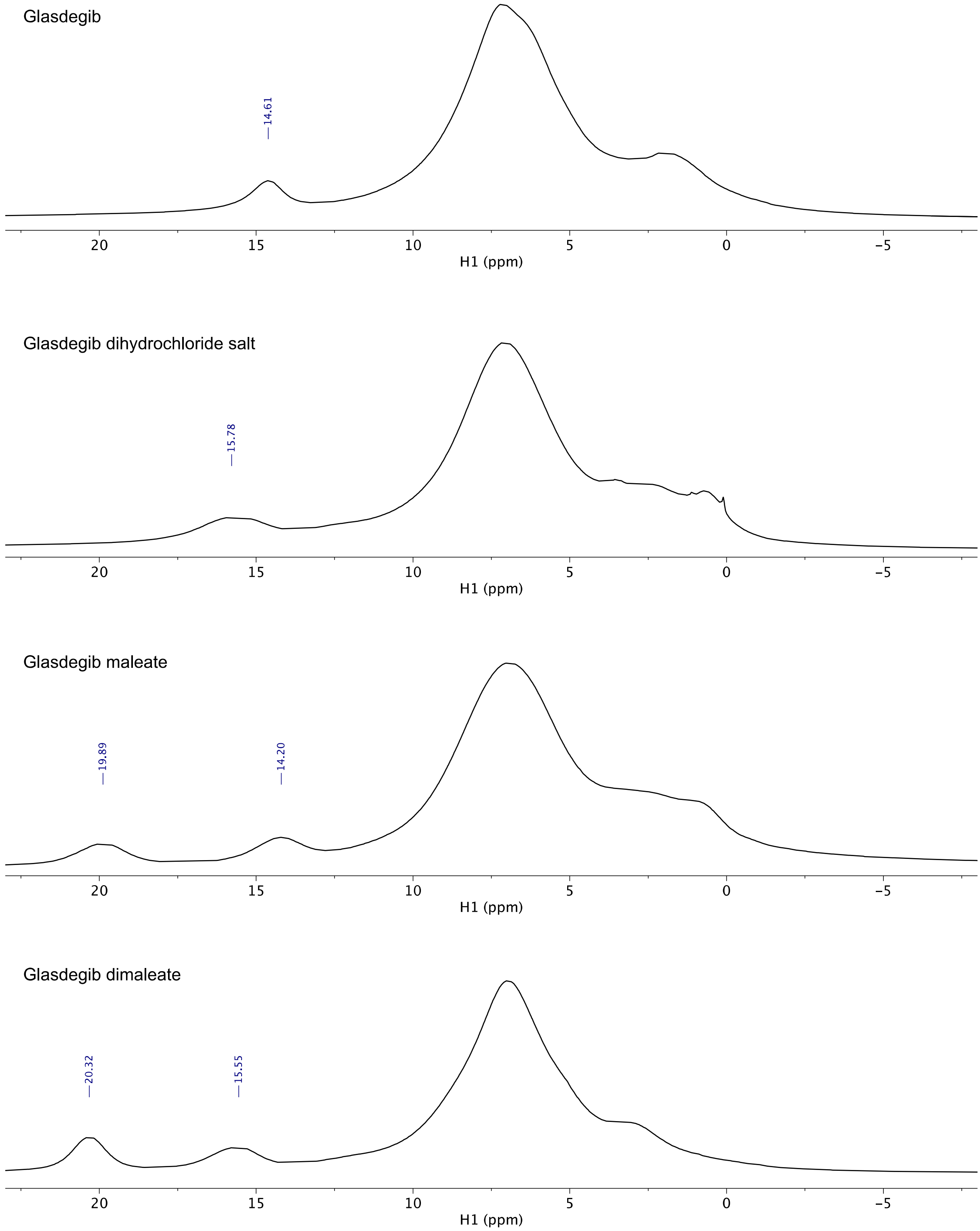

Figure S26. ^{1}H echo MAS NMR spectra of glasdegib base, glasdegib dihydrochloride hydrate, glasdegib monomaleate and glasdegib dimaleate.

7. Solution ^{1}H- and ^{13}C-NMR spectra of glasdegib derivatives

7.1. Glasdegib monomaleate

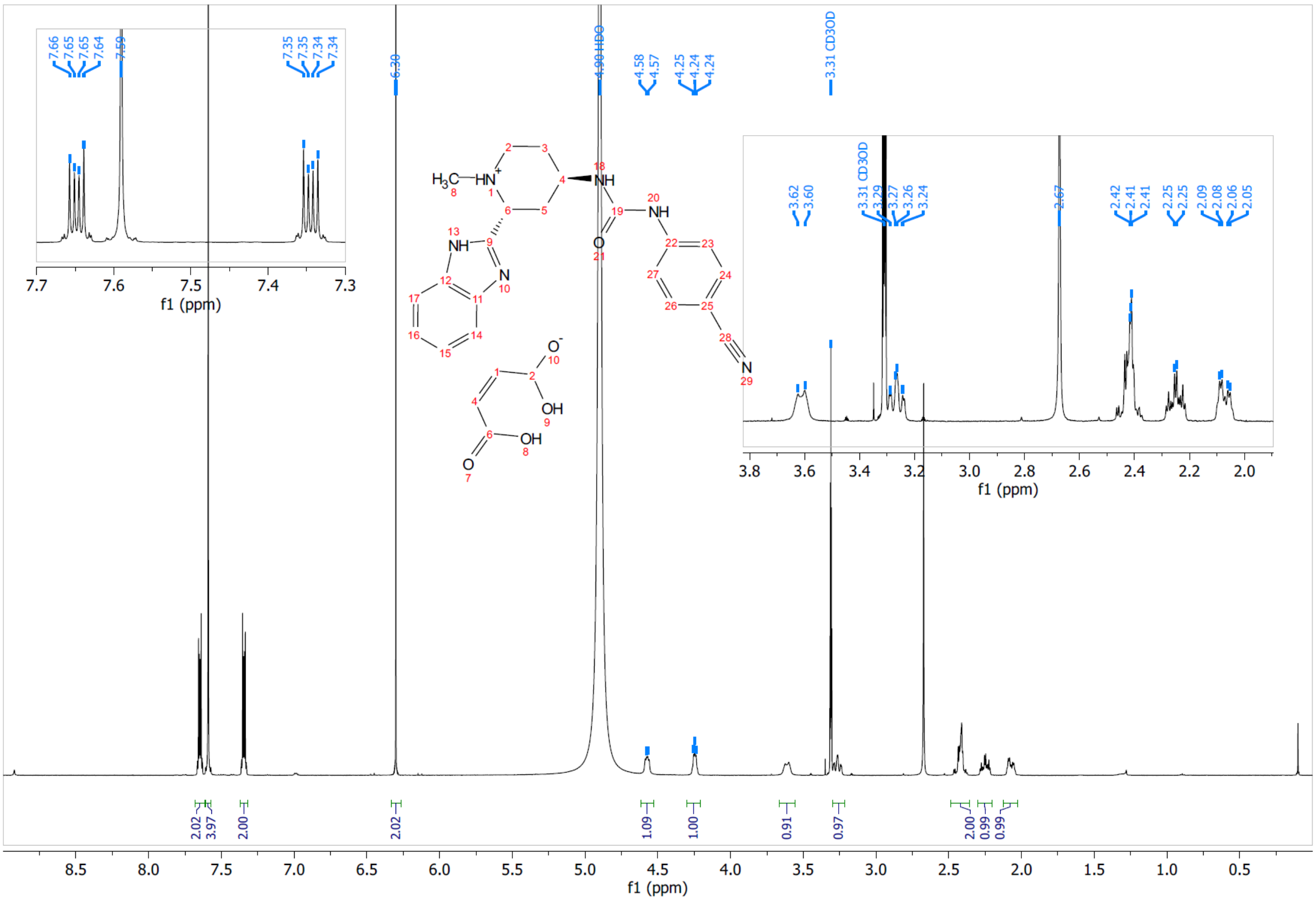

Figure S27. ^{1}H-NMR spectrum of glasdegib monomaleate in MeOD at 500 MHz.

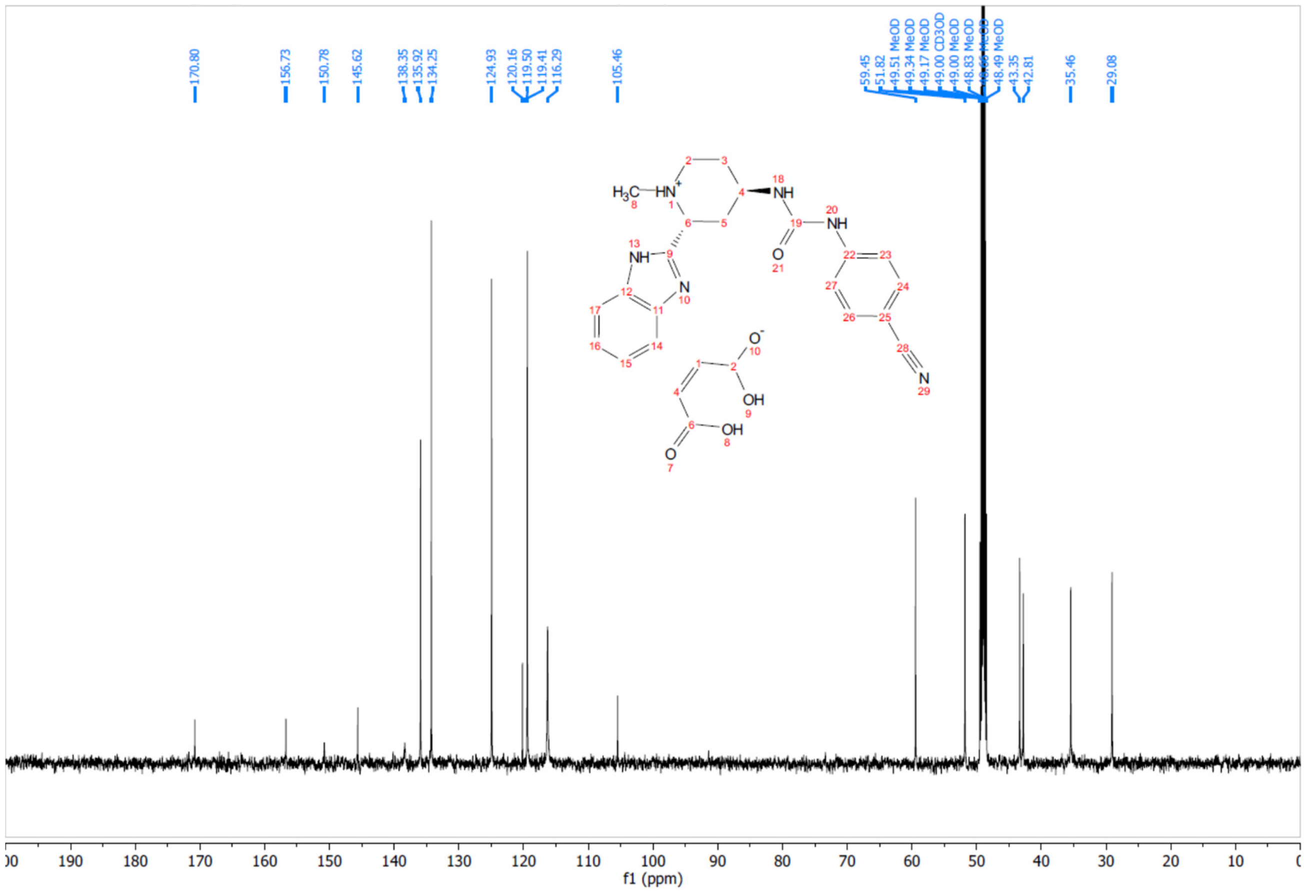

Figure S28. ^{13}C-NMR spectrum of glasdegib monomaleate in MeOD at 125 MHz.

7.2. Glasdegib dimaleate

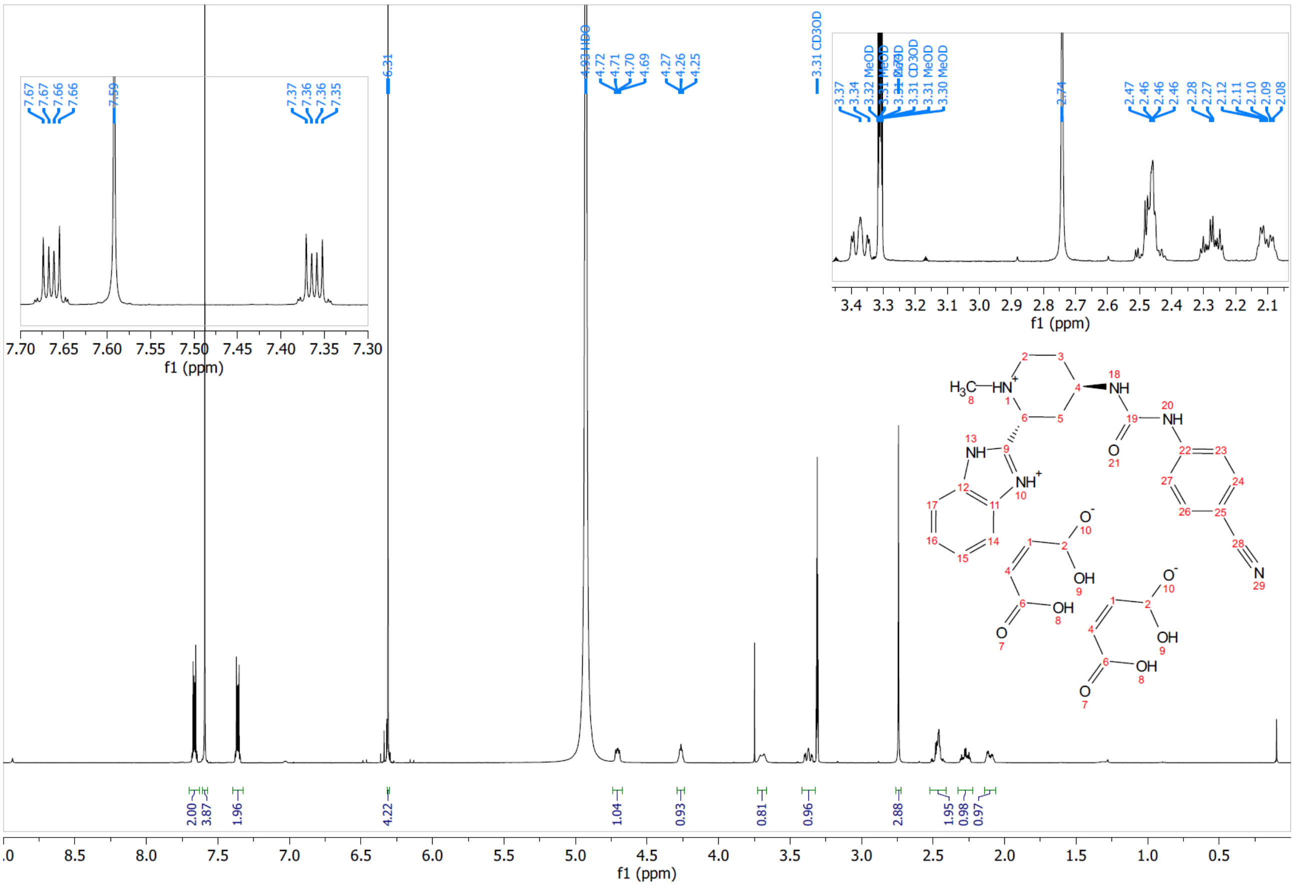

Figure S29. ^{1}H-NMR spectrum of glasdegib dimaleate in MeOD at 500 MHz.

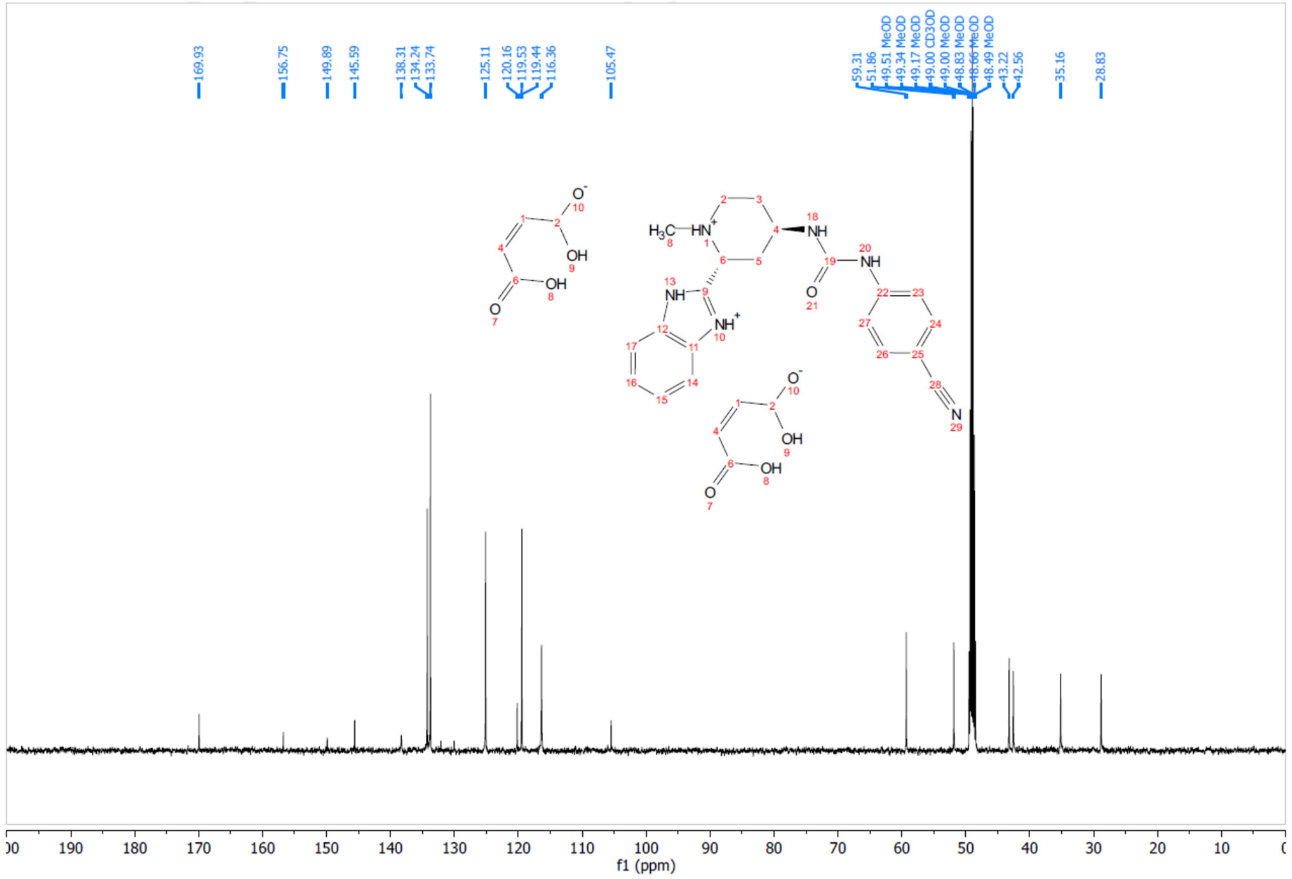

Figure S30. ^{13}C-NMR spectrum of glasdegib dimaleate in MeOD at 125 MHz.

8. Calculated pK_a data

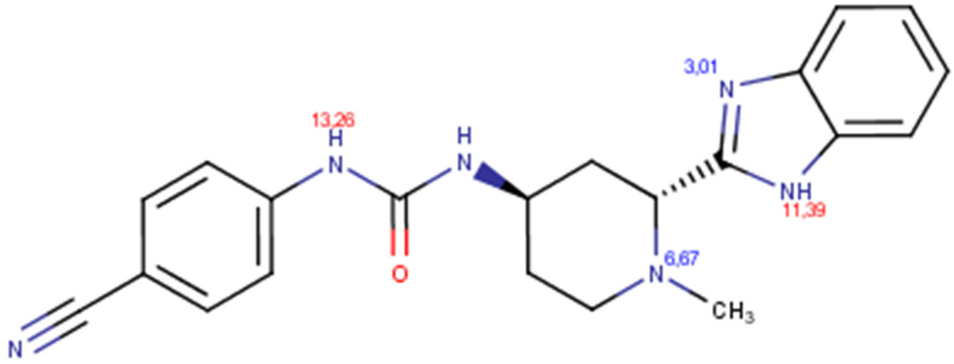

Figure S31. MarvinSketch 17.28 calculated pK_a values for glasdegib.

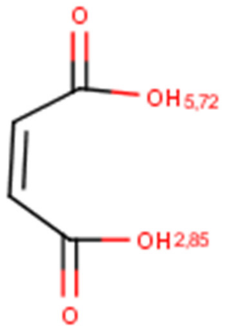

Figure S32. MarvinSketch 17.28 calculated pK_a values for maleic acid.

© 2022 by the author. Licensee MDPI, Basel, Switzerland. This article is an open access article distributed under the terms and conditions of the Creative Commons Attribution (CC BY) license (https://creativecommons.org/licenses/by/4.0/).
